# Oral Collagen Peptides and Skin Rejuvenation: A Systematic Review and an Updated Meta‐Analysis of Randomized Controlled Trials

**DOI:** 10.1111/jocd.71041

**Published:** 2026-07-15

**Authors:** Asia Batool, Muhammad Sharjeel Abbas, Ramzan Farooq, Makhzan Ali Akbar, Alisha Ahmed, Warisha Kanwal, Hamza Ali, Ashfaq Ahmad, Muhammad Junaid, Muhammad Suhaib Hanif, Evardo Barros de Deus Nunes Junior, Hasibullah Aminpoor

**Affiliations:** ^1^ Department of Medicine Sir Syed College of Medical Sciences Karachi Pakistan; ^2^ Department of Medicine, Gomal Medical College Khyber Medical University Peshawar Pakistan; ^3^ Department of Medicine Rawalpindi Medical University Rawalpindi Pakistan; ^4^ Department of Medicine North Sichuan Medical College Nanchong China; ^5^ Department of Medicine Jinnah Sindh Medical University Karachi Pakistan; ^6^ Department of Medicine Karachi Medical and Dental College Karachi Pakistan; ^7^ Department of Medicine Khyber Medical University Peshawar Pakistan; ^8^ Department of Internal Medicine Lady Reading Hospital Peshawar Pakistan; ^9^ Department of Medicine Dow University of Health Sciences Karachi Pakistan; ^10^ Department of Medicine Afya Faculty of Medical Sciences of Pará (FACIMPA) Marabá Brazil; ^11^ Department of Medicine Kabul University of Medical Sciences “Abu Ali Ibn Sina” Kabul Afghanistan

**Keywords:** collagen peptides, hydrolyzed collagen, nutricosmetics, oral collagen, skin aging, skin elasticity, skin hydration, wrinkles

## Abstract

**Background:**

Cutaneous aging is characterized by progressive degradation of the extracellular matrix, manifesting as diminished hydration, loss of elasticity, and increased wrinkle depth. While oral hydrolyzed collagen peptides (HCPs) are increasingly utilized as systemic nutricosmetics, previous evidence syntheses have been limited by severe methodological heterogeneity and the inability to account for highly influential statistical outliers. This updated systematic review and meta‐analysis evaluated the clinical efficacy of oral collagen peptides for skin rejuvenation using an advanced statistical framework.

**Methods:**

A comprehensive literature search was conducted on PubMed (MEDLINE), Cochrane Library, and Google Scholar from inception to November 2025. Randomized controlled trials evaluating oral collagen supplementation in healthy adults were included. Data were synthesized using random effect models. To differentiate true clinical signals from statistical artifacts, analyses were reinforced with multilevel meta‐analysis, leave‐one‐out sensitivity testing, and dose–response spline regressions.

**Results:**

Thirty‐five randomized controlled trials comprising 2534 participants were included. Primary analysis revealed that oral collagen supplementation yielded statistically significant improvements in instrumental measures of skin hydration (SMD = 0.44; 95% CI: 0.15–0.73), skin elasticity (SMD = 0.62; 95% CI: 0.15–1.10), and barrier function via reduced transepidermal water loss (SMD = −0.39; 95% CI: −0.62 to −0.16). Efficacy appeared predominantly time‐dependent rather than dose‐dependent, with elasticity and barrier improvements requiring at least 12 weeks of supplementation. Conversely, rigorous sensitivity and multilevel analyses demonstrated that apparent improvements in structural parameters, specifically skin wrinkles, roughness, and dermal density, were statistical artifacts driven by high‐leverage outliers and severe inter‐study heterogeneity.

**Conclusion:**

Oral collagen peptide supplementation is an effective functional systemic moisturizer that produces significant time‐dependent improvements in cutaneous hydration, elasticity, and barrier retention. However, current evidence from trials of standard duration remains limited and inconsistent regarding structural anti‐aging effects, including macroscopic wrinkle reduction and dermal densification, and the existing data are simply not robust enough to support definitive conclusions in these domains.

**Abbreviations**
BMI: body mass index
CI: confidence intervals
MD: mean difference
MeSH: medical subject headings
PRISMA: preferred reporting items for systematic reviews and meta‐analyses
PROSPERO: prospective register of systematic reviews
RoB 2.0: Cochrane risk of bias tool
SMD: standardized mean difference
TEWL: transepidermal water loss

## 1 Introduction

Skin aging is a multifaceted biological process triggered by the convergence of intrinsic factors, such as cellular senescence, telomere shortening, hormonal decline, and decreased fibroblast activity, and extrinsic factors, such as ultraviolet (UV) radiation, environmental pollution, diet, sleep disorders, and psychological stress. These combined mechanisms activate matrix metalloproteinases (MMPs), enzymes that degrade the extracellular matrix (ECM) and accelerate collagen and elastin breakdown, resulting in the clinical manifestations of wrinkles, loss of firmness, and reduced skin radiance. Visible manifestations of cutaneous aging transcend mere cosmetic concerns; an accumulating body of research indicates that age‐associated alterations in the skin are correlated with diminished self‐esteem, compromised body image, and a decline in overall quality of life, with particularly pronounced effects observed among elderly women [1, 2, 3, 4].

At the molecular centre of this aging process is collagen, the most abundant structural protein in the dermis, which is responsible for tensile strength, elasticity, and hydration of the skin. From the third decade of life onwards, dermal collagen synthesis declines by approximately 1% per year, while the concurrent upregulation of MMP‐1 and MMP‐3 accelerates its fragmentation, producing shorter, disorganized fibers and a progressive reduction in dermal density. Concurrently, hyaluronic acid, a glycosaminoglycan with exceptional water‐binding capacity, is progressively depleted from both the epidermis and dermis, impairing barrier function and driving transepidermal water loss (TEWL). Clinically, these structural alterations present as diminished cutaneous hydration, impaired elastic recoil, pronounced wrinkle formation, and loss of tissue firmness, which together constitute the observable characteristics of skin aging. Although collagen depletion is one of the primary mechanisms driving aging of the skin, it is important to note that skin aging is a complex process involving multiple factors such as cumulative UV damage, hormonal changes especially around the menopause, intrinsic chronological aging, and modifiable lifestyle factors including smoking, diet and sleep, which act in concert, but not individually. It is therefore important to consider the overall biology when assessing any intervention for its ability to contribute to skin aging [3, 5].

In response to the growing consumer demand for endogenous skin restoration, the field of nutricosmetics, which involves ingestible bioactive compounds designed to improve skin health from within, has expanded substantially [6], with the global market projected to grow at a compound annual growth rate (CAGR) of over 8% by 2031. Among the most studied nutricosmetic ingredients are oral hydrolyzed collagen peptides (HCPs), low‐molecular‐weight bioactive fragments derived from bovine, porcine, marine, or novel sources that are engineered for high gastrointestinal bioavailability. Unlike intact collagen, these dipeptides and tripeptides, specifically prolyl‐hydroxyproline (Pro‐Hyp) and hydroxyprolyl‐glycine (Hyp‐Gly), are partially absorbed in their intact forms into the systemic circulation and subsequently accumulate within the dermal layer. They are proposed to stimulate the activity of fibroblasts which in turn stimulates the synthesis of collagen and glycosaminoglycans; this has been demonstrated in vitro using cultured human dermal fibroblasts; however, this has not been fully established in vivo in the clinic [7, 8].

Over the past decade, randomized controlled trials (RCTs) have progressively characterized the dermatological efficacy of HCPs across multiple parameters. Bovine‐derived bioactive collagen peptides enriched with Pro‐Hyp have demonstrated significant improvements in facial moisture, elasticity (R2 parameter), and wrinkle reduction in double‐blind, placebo‐controlled studies. Marine‐sourced collagen peptides, including those from freshwater fish varieties, have been shown to reduce skin wrinkle scores and significantly improve cheek elasticity, particularly in peri‐menopausal women, a population especially vulnerable to accelerated collagen loss. The combination of collagen with coenzyme Q10 (CoQ10), a potent mitochondrial antioxidant, has improved the periorbital wrinkle area and dermis density, adding an oxidative protection dimension to the structural benefits of peptide supplementation. Specific bioactive collagen peptides of bovine origin have been demonstrated to diminish ocular wrinkle volume while markedly increasing dermal levels of elastin and procollagen type I, thereby substantiating their role in restoring the extracellular matrix framework [9, 10, 11, 12].

Oral HCPs are more versatile than isolated skin effects, and there is more evidence supporting this claim. Collagen combined with bio‐active substances such as coenzyme Q10 has demonstrated beneficial effects on structural and oxidative stress parameters of the aging of the skin. Likewise, the collagen peptides derived from fish, such as tuna, have been effective in several skin parameters in RCTs, and the effects have been maintained for as long as 12 months after the final dose. The results of these studies together indicate that the effectiveness of oral collagen could be applicable to a variety of formulations and demographics, and warrant a more comprehensive, up‐to‐date review of the evidence [13, 14, 15].

Regardless of this, there are gaps in the literature. First, there have been inconsistent definitions for outcomes in the previously conducted systematic reviews and meta‐analyses, making it difficult to have confidence in pooled estimates because of heterogeneity in peptide sources and molecular weights, heterogeneous dosing regimens, follow‐up periods that were short, and high percentages of industry‐funded trials. Second, and more significantly, there are significant differences in the collagen formulations and doses used in the various published studies, and it is not known whether there is a dose response effect, or if there is a threshold dose, so that increases in collagen beyond this point would be without further benefit. Third, to date no meta‐analysis has adopted a multilevel analysis to separate the effects of peptide source, molecular weight, duration of supplementation, and type of formulation from specific skin outcome parameters. Some of the important points noted are the findings of Myung and Park (2025), which showed that significant effects were not found if only independent or high quality trials were included in the analyses, highlighting the need for a more rigorous and bias‐informed synthesis. The present study was aimed towards directly filling these gaps [16, 17].

This updated systematic review and meta‐analysis addresses these critical gaps by synthesizing the most recent randomized controlled trials utilizing a highly rigorous analytical framework. The primary objective of this study was to provide precise and updated clinical evidence on the efficacy of oral collagen peptides in skin rejuvenation. By employing advanced statistical techniques, including dose–response spline regressions, multilevel meta‐analysis, leave‐one‐out sensitivity testing, and contour‐enhanced funnel plots, this study aimed to differentiate true physiological improvements from statistical artifacts across key dermatological parameters, such as hydration, elasticity, transepidermal water loss (TEWL), wrinkles, and dermal density.

## 2 Methods

### 2.1 Study Design and Reporting Guidelines

**FIGURE 1:**
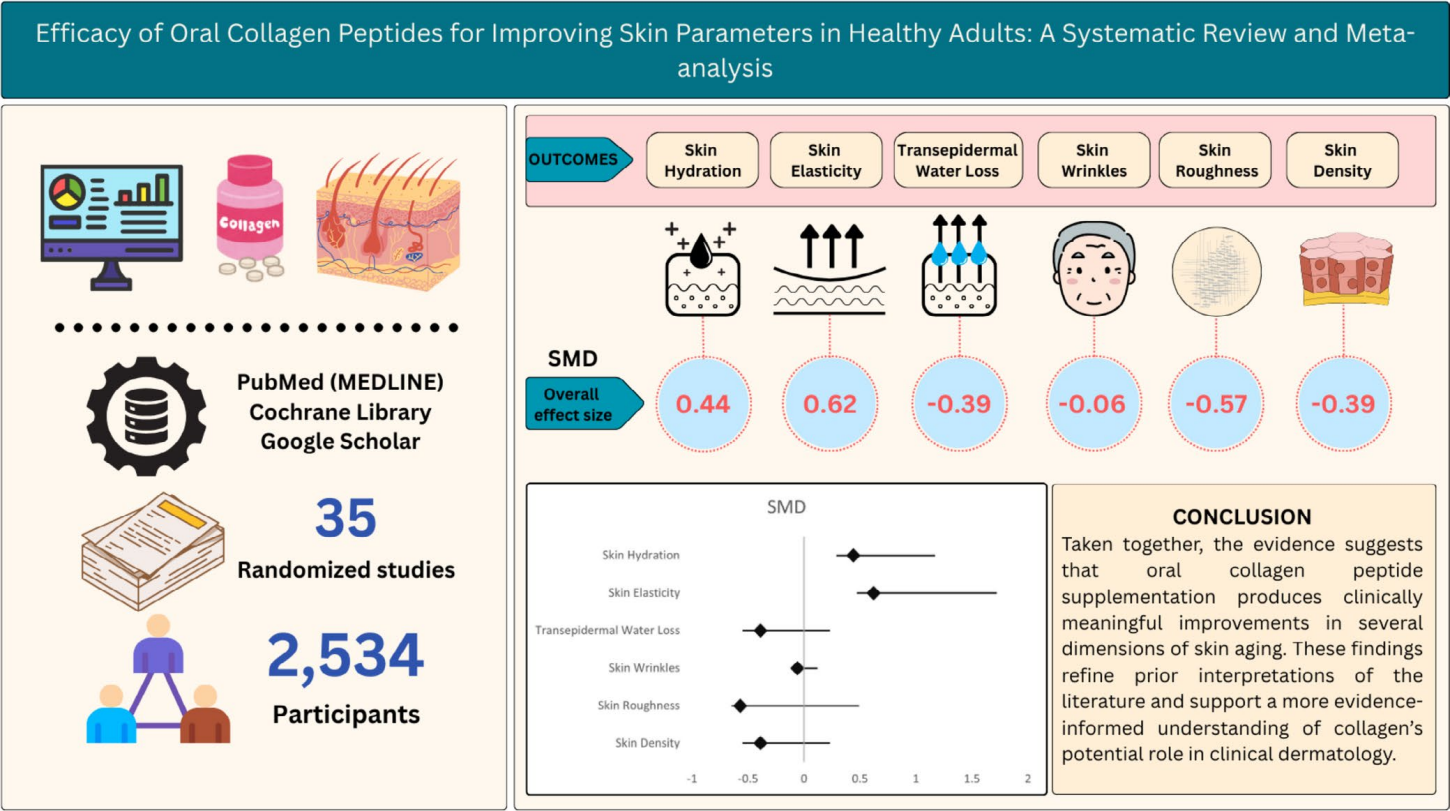
Graphical abstract for a systematic review and meta‐analysis evaluating the efficacy of oral collagen peptides for improving skin parameters in healthy adults.

This systematic review and meta‐analysis was conducted in accordance with the Preferred Reporting Items for Systematic Reviews and Meta‐Analyses (PRISMA) 2020 guidelines, ensuring transparency, methodological rigor, and reproducibility [18, 19]. The protocol is registered with the International Prospective Register of Systematic Reviews (PROSPERO) (registration details withheld for blinded review) (Figure 1).

### 2.2 Literature Search Strategy

A comprehensive literature search was performed in the PubMed (MEDLINE), Cochrane Library, and Google Scholar databases from inception to November 2025, with the final search executed on November 30, 2025. The search strategy combined Medical Subject Headings (MeSH) and free‐text keywords related to collagen supplementation and skin health, including “collagen peptides,” “hydrolyzed collagen,” “nutricosmetics,” “oral collagen,” “skin aging,” “skin hydration,” “skin elasticity,” and “wrinkles.” Boolean operators (“AND”, “OR”) were applied to refine the search. The reference lists of all included studies and relevant systematic reviews were manually screened to identify any additional eligible studies not captured through the electronic search. Regarding gray literature, conference abstracts, unpublished trials, and non‐peer‐reviewed reports were not systematically searched; however, Google Scholar was included as a supplementary database to broaden retrieval beyond indexed sources. The detailed search strategy is presented in the Supporting Information Appendix page 6–7.

### 2.3 Study Selection Process

All retrieved records were imported into reference management software, and duplicate studies were excluded. Two independent reviewers screened the titles and abstracts for relevance. Full‐text articles were retrieved for studies that met the inclusion criteria or for which eligibility was unclear. Any discrepancies between reviewers were resolved through discussion and consensus, with a third author consulted when necessary. The study selection process is summarized in the PRISMA 2020 flow diagram.

### 2.4 Inclusion Criteria

Studies were included if they met the following criteria: (1) human randomized controlled trials or controlled clinical studies; (2) oral administration of collagen peptides or collagen‐based nutricosmetic supplements; (3) presence of a placebo or control group; (4) assessment of skin‐related outcomes, such as hydration, elasticity, wrinkle depth, skin thickness, or dermal collagen density; and (5) availability of sufficient quantitative data for meta‐analysis.

### 2.5 Exclusion Criteria

Studies were excluded if they met any of the following criteria: (1) evaluated topical collagen administration or cosmetic products without an oral intervention; (2) interventions where oral collagen was heavily confounded by the co‐administration of other active aesthetic ingredients (e.g., vitamins, hyaluronic acid, or other botanical extracts) unless the effects could be statistically isolated; (3) evaluated participants with diagnosed dermatological diseases (e.g., psoriasis, atopic dermatitis) rather than populations focused on healthy skin aging; (4) comprised in vitro, ex vivo, or animal model studies; and (5) non‐original research, including review articles, editorials, conference abstracts, or trial protocols.

### 2.6 Data Extraction

Data extraction was conducted independently by two authors using a standardized data extraction form. The extracted information included author name, year of publication, country, study design, sample size, participant demographics, type and source of collagen, dosage, duration of supplementation, outcome measures related to skin aging, and main results. Any discrepancies in the extracted data were resolved by consensus.

### 2.7 Quality Assessment

**FIGURE 2:**
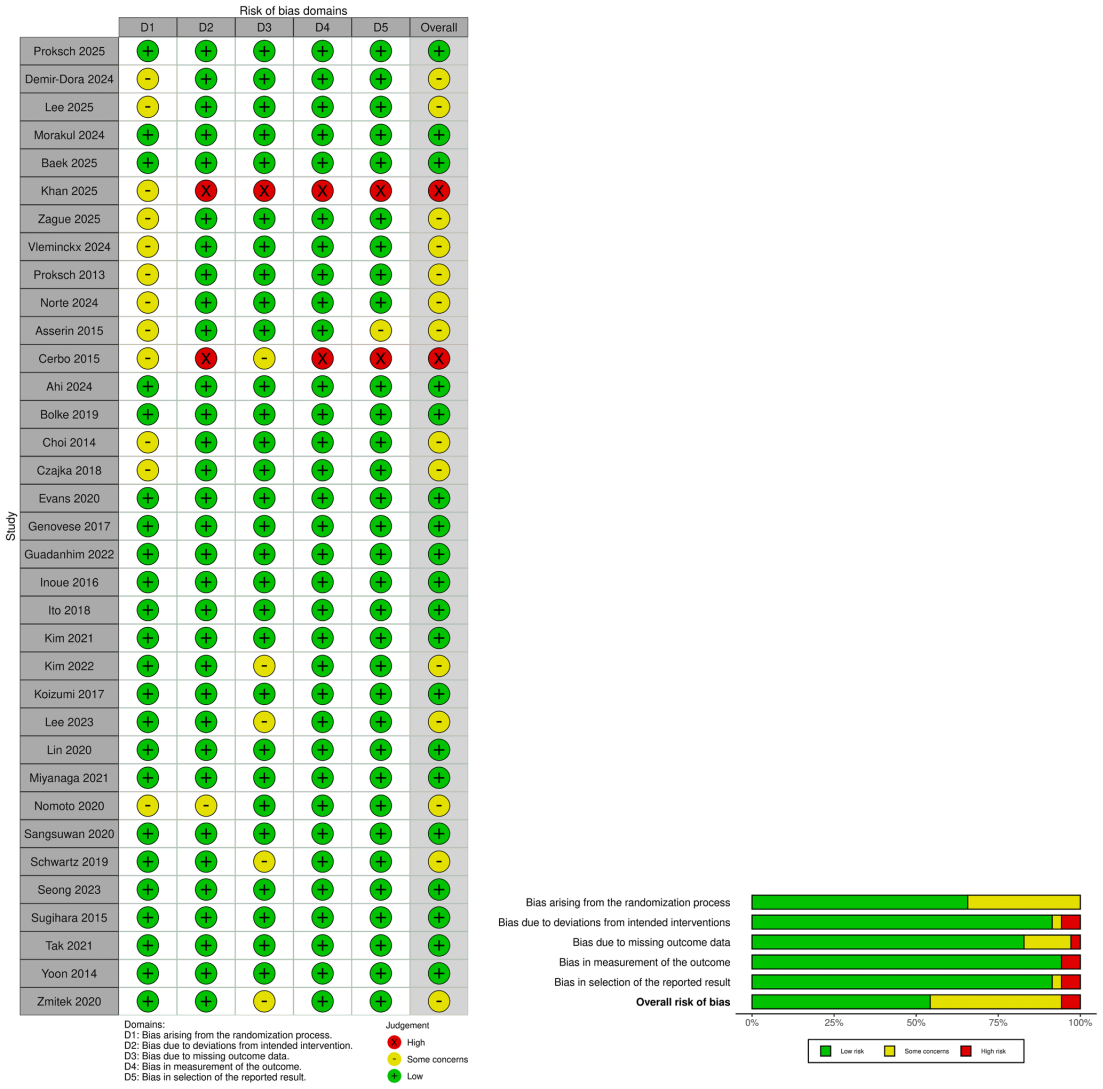
Traffic plot for Cochrane Risk of Bias (RoB 2.0) assessment. Red is for high risk, yellow is for moderate risk and gren is for low risk of bias.

The methodological quality and risk of bias of the included randomized controlled trials were assessed using the Cochrane Risk of Bias tool (RoB 2.0). The domains evaluated included random sequence generation, allocation concealment, blinding of participants and outcome assessors, incomplete outcome data, selective reporting, and other potential sources of bias [20]. Each study was categorized as having a low, some concerns, or high risk of bias (Figure 2).

### 2.8 Outcome Measures

The primary outcomes included objective measures of skin hydration, elasticity, wrinkle depth or number, and dermal collagen content. Secondary outcomes included skin thickness, Transepidermal Water Loss (TEWL), and reported adverse events associated with collagen supplementation.

### 2.9 Statistical Analysis

All statistical analyses were performed using the R statistical software (version 4.3.3) [21]. A meta‐analysis was performed using a random‐effects model to account for clinical and methodological heterogeneity among the studies. Continuous outcomes were summarized using the mean difference (MD) or standardized mean difference (SMD) with 95% confidence intervals (CI). Statistical heterogeneity was assessed using the *I* ^2^ statistic, with values greater than 50% indicating substantial heterogeneity. Sensitivity analyses were conducted to evaluate the stability of the pooled estimates by excluding individual studies. Publication bias was assessed using funnel plot analysis when sufficient studies were available.

Where possible, subgroup analyses and meta‐regression were used to examine potential sources of between‐study variability in the collagen interventions used, such as the form of collagen (e.g., bovine collagen, marine collagen, and fish collagen), the molecular weight of the collagen, dose (ranging from 500 mg/day to 10 g/day), and formulation (e.g., collagen alone, collagen plus other bioactive ingredients). This formulation heterogeneity is recognized as an intrinsic weakness of the existing evidence‐base and could account for the considerable statistical heterogeneity seen across outcome domains.

## 3 Results

### 3.1 Study Selection & Baseline Demographics

**FIGURE 3:**
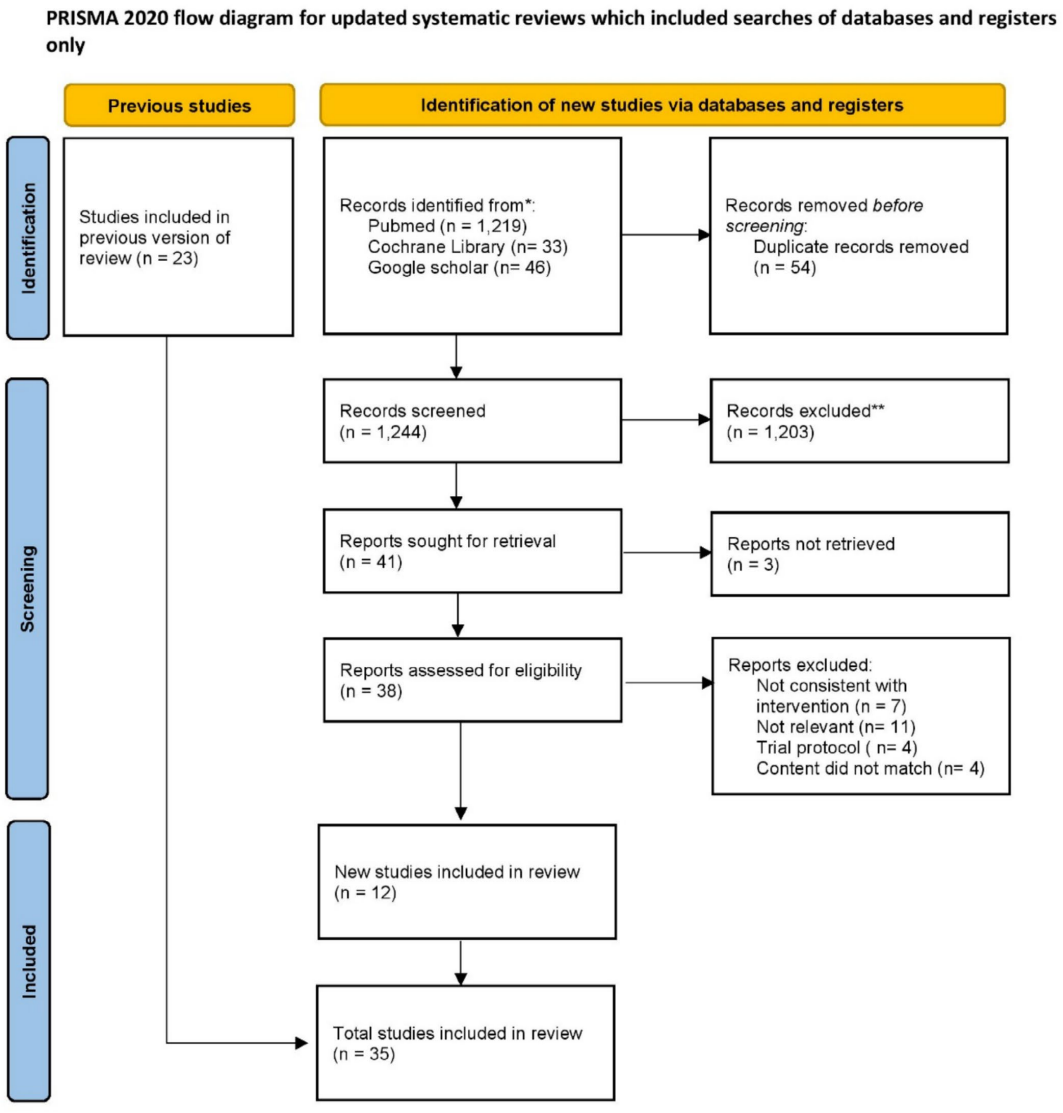
PRISMA flowchart.

A total of 1298 records were identified through database searches. After the removal of duplicates, 1244 records were screened. Following title and abstract screening, 1203 records were excluded from the study. Forty‐one full‐text articles were assessed for eligibility, and 35 studies met the inclusion criteria and were included in the final meta‐analysis, as shown in the PRISMA 2020 flow diagram in Figure 3. A total of 35 randomized controlled trials were included in the analysis. Most studies were conducted in South Korea, followed by Japan, China, Thailand, and Taiwan. Daily collagen doses varied considerably across included studies, ranging from a minimum of 500 mg to a maximum of 10 g, representing a 20‐fold difference between the lowest and highest doses. Collagen sources included bovine, fish/marine, and specialized tripeptide preparations. Intervention durations ranged from 4 to 16 weeks, and most of the studies included had a 12 week treatment regimen. However, it should be noted that the duration of these supplements may not be long enough to detect any meaningful dermal remodeling or macroscopic improvements, such as wrinkle reduction, which likely require longer supplementation periods. 2534 participants were included and the population sample was predominantly female (15 of the 35 studies only recruited female cohorts). The participants were mostly middle‐aged, with mean ages typically ranging between 40 and 60 years old, but the age spanned to older adults, with a maximum age of 82 years. Additional baseline characteristics are presented in Table 1.

**TABLE 1:** Baseline characteristics of included studies.

Study ID	Country	Population (*N*)		Gender (F/M)		Age (mean ± SD)	Collagen type/dose (per day)	Measured outcomes	Follow‐up (weeks)
		Intervention (*N*)	Control (*N*)	Intervention	Control				
Baek (2025) [22]	Switzerland	49	49	29/21	28/22	43.4 ± 6.0	1.5 g Plant‐based Alternative	Skin Hydration, TEWL, Skin Moisture, Keratin Index, Skin Elasticity, Periorbital Wrinkles, Skin Texture	12
Khan (2025) [23]	Pakistan	28	27	27/7	20/7	42.5 ± 8.2	10 g Hydrolysed	Skin Hydration, Wrinkle Depth, Fine Lines, Skin Elasticity, Skin Firmness	12
Lee (2025) [24]	South Korea	35	35	33/2	33/2	46.7 ± 9.8	1650 mg LMW	Wrinkle Assessment, Skin Elasticity, Skin Density, Skin Hydration, Pores, Sebum, Stratum Corneum	10
Proksch (2025) [25]	Germany	33	33	33/0	33/0	45.8 ± 5.8	2.5 g Bioactive (SCPs) type 1	Skin Wrinkles, Elasticity, Hydration	8
Zague (2025) [26]	Brazil	42	43	42/0	43/0	52.5 ± 2.5	2.5 g Col‐OP	Skin Hydration, Firmness, Elasticity	12
Ahi (2024) [27]	Turkey	36	36	36/0	36/0	52.5 ± 4.2	10 g Hydrolysed	Skin Elasticity Measurements, Skin Hydration, TEWL, Skin Wrinkling Count Measurement	12
Demir‐Dora (2024) [28]	Turkey	57	55	57/0	55/0	44.4 ± 5.9	10 g Type 1 & 3 Peptides	Skin Elasticity, Skin Hydration, Skin Roughness	12
Morakul (2024) [14]	Thailand	36	36	36/0	36/0	50.0 ± 3.3	5 g Peptides (Tuna)	Skin Hydration, Skin Density, Trans epidermal Water Loss (TEWL), Skin Elasticity	10
Seong (2024) [29]	Spain	40	40	40/0	40/0	46 ± 7.4	2.5 g LMW Bovine Peptides (Type I & III)	Facial Wrinkle Parameters (volume, area, depth), Skin Hydration, Skin Elasticity	6
Vleminckx (2024) [30]	China	43	42	43/0	42/0	54.0 ± 3.7	5 g Porcine Skin‐derived Peptides	Dermis Density, Skin Moisture, Elasticity, Wrinkle Visibility, Nail Color and Health, Skin Beauty Perception, Skin and Nail Aging Signs	12
Lee (2023) [31]	South Korea	54	46	54/0	46/0	45.3 ± 6.3	1650 mg LMW Fish‐derived Peptides	Skin Hydration, Desquamation (skin shedding), Skin Wrinkling, Skin Elasticity	12
Seong (2024) [29]	South Korea	45	42	33/12	30/12	44.5 ± 6.2	2 g LMWCP	Skin Hydration, Skin Elasticity, Skin Wrinkles, Skin Whitening	12
Guadanhim (2023) [32]	Brazil	14	14	14/0	14/0	69.5 ± 7.3	5 g oral HC + 2.5% Topical HCt	Skin Elasticity, Skin Echogenicity, Skin Thickness	25
Kim (2022) [33]	South Korea	43	41	40/3	36/5	49.4 ± 6.6	500 mg LMWCP	Skin Wrinkles, Skin Elasticity, Skin Hydration	12
Kim (2018) [34]	South Korea	33	31	33/0	31/0	48.2 ± 4.4	1 g LMWCP	Skin Hydration, Visual assessment of crow's feet score, Skin Wrinkling Parameters, Skin Elasticity	12
Miyanaga (2021) [35]	Japan	31 (1 g)33 (5 g)	33	31/0 (1 g)33/0 (5 g)	33/0	43.0 ± 4.0	1 g 5gFish‐Derived Peptides	Stratum corneum (SC) Water Content, Epidermal Water Content, Dermal Water Content, TEWL, Skin Elasticity, Skin Thickness, NMF Components (PCA, UCA, amino acids)	12
Tak (2021) [36]	South Korea	42	42	42/0	42/0	48.0 ± 5.9	1 g Tripeptide (Fish)	TEWL, Skin Hydration, Skin Elasticity, Skin Wrinkles	12
Evans (2021) [10]	Canada	17	19	17/0	19/0	54.4 ± 3.4	10 g Fish Derived	Skin Wrinkles, Skin Elasticity, Firmness Score, Radiance Score, Wrinkle Score	12
Lin (2023) [15]	Taiwan	25	25	25/0	25/0	42.5 ± 3.8	50 mL drink containing: 11% fish collagen 2% Djulis extract	Skin hydration, Skin Brightness, Skin Wrinkles, Skin Texture, Skin Pores, Skin Spots, Collagen Content	8
Nomoto (2020) [37]	Japan	20	19	14/6	13/6	80.5 ± 6.8	10 g Peptides	Stratum Corneum Hydration, Skin Elasticity	8
Sangsuwan (2021) [38]	Thailand	17	19	17/0	19/0	56.1 ± 2.8	5 g Hydrolysed	Skin Elasticity	8
Zmitek (2020) [39]	Slovenia	16	15	16/0	15/0	54.6 ± 6.9	4 g Hydrolysed (Fish)	Dermis Density & Thickness, Skin Elasticity, Skin Hydration, TEWL, Periorbital Wrinkle Area Fraction, Total Wrinkle Score (TWS), Skin Smoothness & Microrelief	12
Bolke (2019) [40]	Germany	36	36	36/0	36/0	54.0 ± 6.3	2.5 g Peptides	Roughness, Skin Density, Skin Elasticity, Skin Hydration	16
Schwartz (2019) [41]	USA	58	55	58/0	55/0	50.9 ± 5.4	1000 mg per dose containing:≥ 600 mg hydrolysed type II ≥ 200 mg chondroitin sulfate ≥ 100 mg hyaluronic acid	TEWL, Skin Hydration, Skin Elasticity, Collagen Content, Wrinkles (crow's feet and global), Texture/Smoothness, Skin Tone, Melanin, Hemoglobin, Dryness, Erythema	12
Czajka (2018) [42]	Italy	61	59	50/11	41/18	43.0 ± 12.7	4 g Hydrolysed (Fish)	Skin Elasticity	12
Genovese (2017) [43]	Italy	60	60	57/3	54/6	48.7 ± 6.5	5 g Hydrolysed type 1	Skin Elasticity	12
Koizumi (2017) [44]	South Korea	37	34	37/0	34/0	46.9 ± 5.0	3 g Peptides (Fish)	Periorbital Wrinkles, Skin Elasticity, Facial Skin Moisture, Blood Parameters	12
Inoue (2016) [45]	China	28 (L‐CP)26 (H‐CP)	26	28/0 (L‐CP)26/0 (H‐CP)	26/0	42.6 ± 4.6	5 g 0.1 g per kg of product (L‐CP)2 g kg of product (H‐CP)Hydrolysed (Fish)	Facial Skin Wrinkles and Roughness, Facial Skin Moisture, Facial Skin Elasticity, Blood Parameters	8
Cerbo (2015) [13]	Italy	15	15	15/0	15/0	43.6 ± 1.2	248 mg Hydrolysed (Fish)	Facial Sebum, Skin Hydration, Skin Tonicity, Skin Elasticity, Facial Skin pH, Fibronectin, Hyaluronic Acid, Neutrophil Elastase 2, Elastin, Carbonylated Proteins, Photoaging Severity (VAS)	6
Sugihara (2015) [46]	China	27	26	27/0	26/0	42.8 ± 4.7	2.5 g Hydrolysed	Facial Skin Hydration, Facial Skin Elasticity, Facial Skin Roughness	8
Choi (2014) [47]	South Korea	8	8	6/2	6/2	35.5 ± 4.0	3 g 15% Tripeptide	Skin Hydration, Trans epidermal Water Loss (TEWL), Skin Elasticity, Erythema Index (EI), Melanin Index (MI)	12
Yoon (2014) [48]	South Korea	22	22	22/0	22/0	51.1 ± 5.3	3 g Hydrolysed	Elasticity, Hydration, TEWL, Molecular Biomarkers (procollagen mRNA, MMP‐1, MMP‐12), UV‐induced DNA damage (thymine dimers, 8‐OHdG)	12
Proksch (2014) [49]	Germany	57	57	57/0	57/0	55.6 ± 6.0	2.5 g Bioactive Peptides	Eye Wrinkle Volume, Content of Type I Procollagen, Elastin, and Fibrillin in Skin Fluid	12

### 3.2 Primary Endpoints

#### 3.2.1 Skin Hydration

**FIGURE 4:**
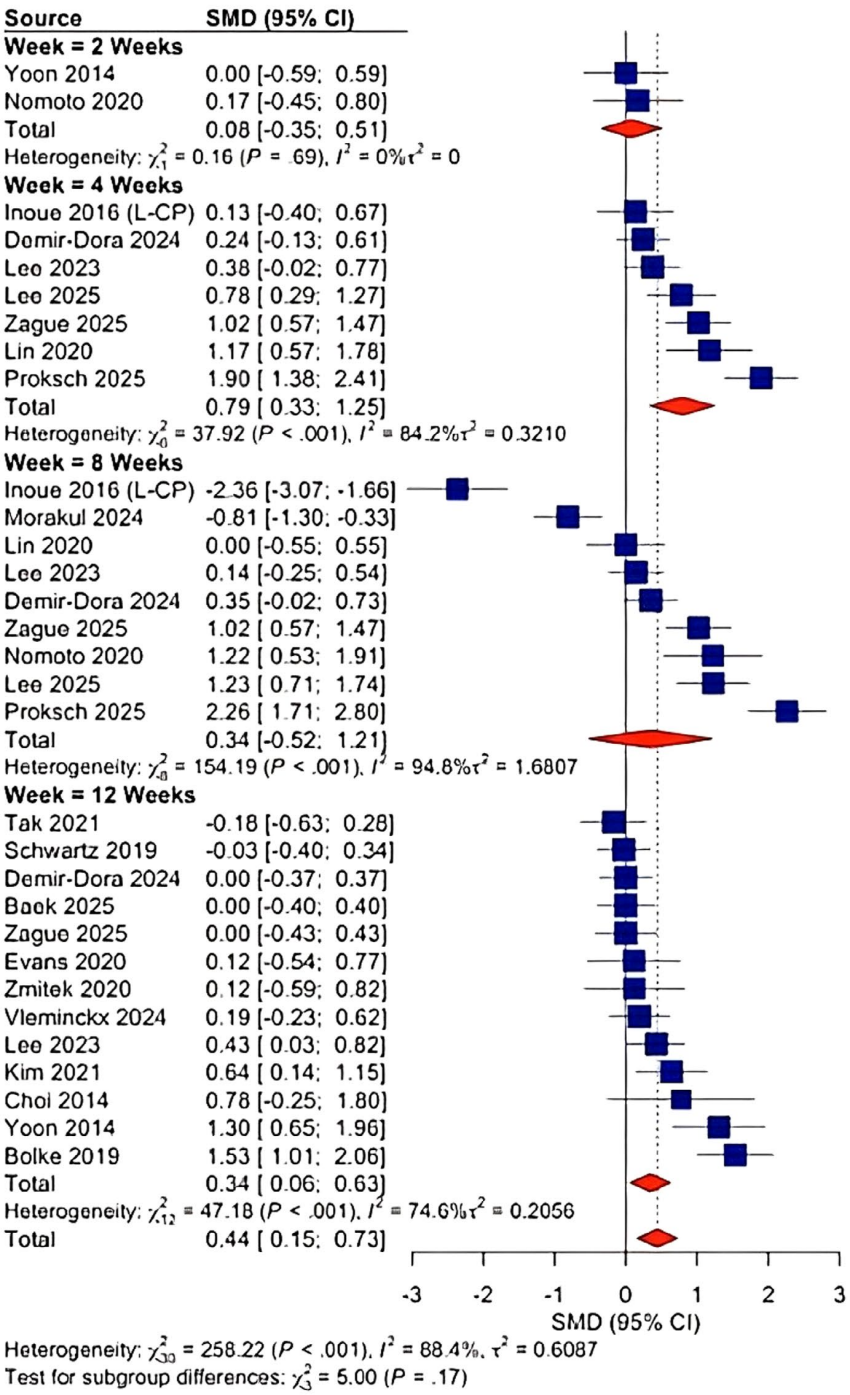
Forest plot showing standardized mean difference (SMD) with 95% confidence intervals (CIs) comparing the intervention group with controls in different time stamps for skin hydration.

The primary analysis demonstrated an overall standard mean deviation (SMD = 0.44, 95% CI: 0.15–0.73; Figure 4), with significant improvement at week 12 (SMD = 0.34, 95% CI: 0.06–0.63; Figure 4) and an even stronger effect at week 4 (SMD = 0.79, 95% CI: 0.33–1.25; Figure 4), resulting in a cumulative overall effect size of approximately 0.44. The primary analysis demonstrated a statistically significant overall improvement following the exclusion of extreme outliers (Morioka 2024, Koizumi 2017), which initially inflated the heterogeneity to an *I* ^2^ of 93%. Efficacy was notably time‐dependent, with significant improvements at Weeks 4 and 12, while intermediate time points (Week 8 SMD = 0.34, 95% CI = −0.53 to 1.21; Figure 4) failed to reach significance.

#### 3.2.2 Skin Elasticity

**FIGURE 5:**
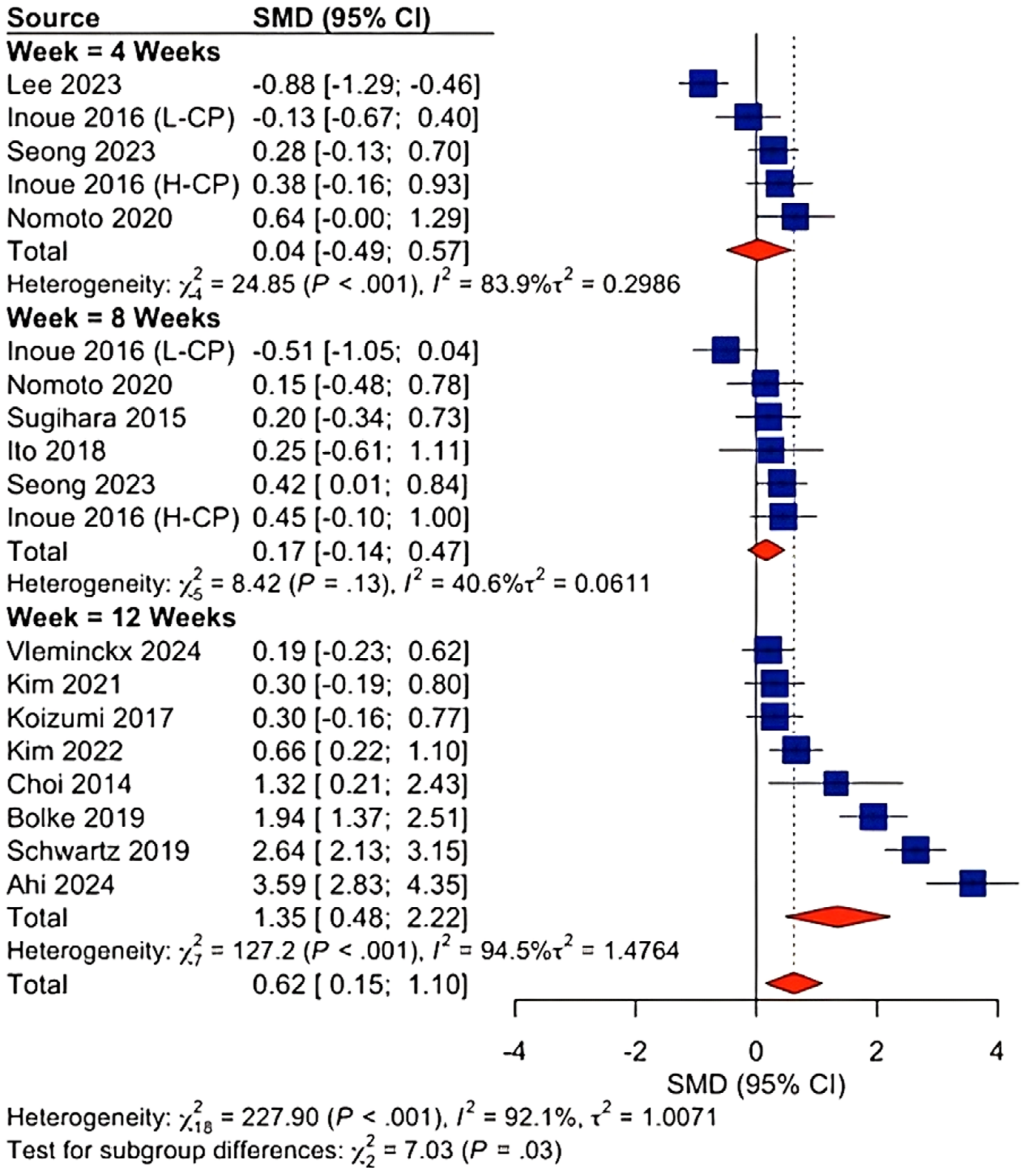
Forest plot showing standardized mean differences (SMD) with 95% confidence intervals (CIs) comparing the intervention group with controls in different time stamps for skin elasticity.

The initial assessment of skin elasticity suggested a lack of therapeutic benefit (SMD = −0.00, 95% CI: −0.99 to 0.98, *p* = 0.87; Figure S2A), a finding heavily distorted by two extreme negative outliers at week 12 [29, 39]. Following the exclusion of these high‐leverage studies, the corrected meta‐analysis revealed a statistically significant overall improvement (SMD = 0.62, 95% CI: 0.15–1.10, *p* = 0.03; Figure 5). Efficacy appeared predominantly time‐dependent, with meaningful improvements emerging most consistently at later time points. Short‐term supplementation yielded negligible results, with no significant improvement observed at week 4 (SMD 0.04, 95% CI = −0.49 to 0.57; Figure 5) or week 8 (SMD 0.17; 95% −0.14 to 0.47; Figure 5). A robust therapeutic effect emerged exclusively in the Week 12 subgroup, which demonstrated a large effect size of 1.35 (95% CI: 0.48–2.22; Figure 5). Despite the correction, heterogeneity remained substantial in the long‐term cohort (*I* ^2^ = 92.1%, *τ* ^2^ = 1.47), indicating that while the intervention is effective on average after week 12, the magnitude of participant response varies significantly across trials.

#### 3.2.3 Transepidermal Water Loss (TEWL)

**FIGURE 6:**
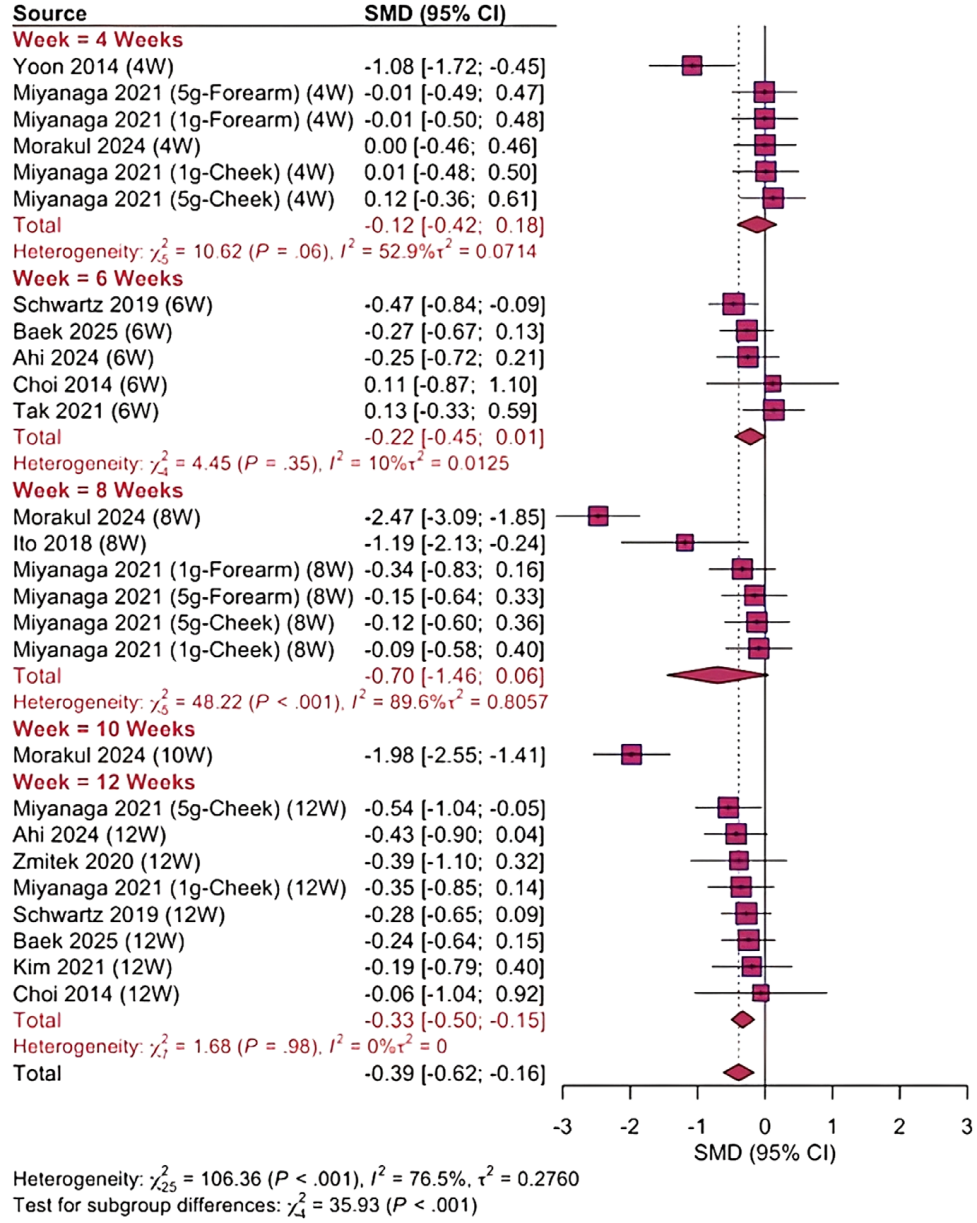
Forest plot showing standardized mean differences (SMD) with 95% confidence intervals (CIs) comparing the intervention group with controls in different time stamps for transepidermal water loss (TEWL).

The analysis supported a barrier‐strengthening effect, showing a statistically significant reduction in water loss (SMD = −0.39, 95% CI: −0.62 to −0.16, *p* < 0.001; Figure 6). The therapeutic efficacy was strictly time‐dependent. Short‐term supplementation yielded no distinguishable benefit over placebo at Week 4 (SMD = −0.12, 95% CI = −0.42 to 0.18; Figure 6), Week 6 (SMD = −0.22, 95% CI = −0.45 to 0.01; Figure 6), and Week 8 (SMD = −0.70, 95% CI = −1.46 to 0.06; Figure 6). A robust therapeutic effect appeared predominantly time‐dependent, with meaningful improvements emerging most consistently at later time points. Week 10 (SMD = −1.98, 95% CI = −2.55 to −1.41; Figure 6) and Week 12 cohorts (SMD = −0.33, 95% CI: −0.50 to −0.15; Figure 6). Notably, the aggregate estimate was heavily compromised by a single extreme outlier at week 10, Morakul 2024 (SMD = −1.98), which artificially inflated the pooled effect size. Heterogeneity remained high throughout (*I* ^2^ = 91.8%), indicating that while the barrier improved on average, the magnitude of that improvement varied significantly between individuals.

#### 3.2.4 Skin Wrinkles

**FIGURE 7:**
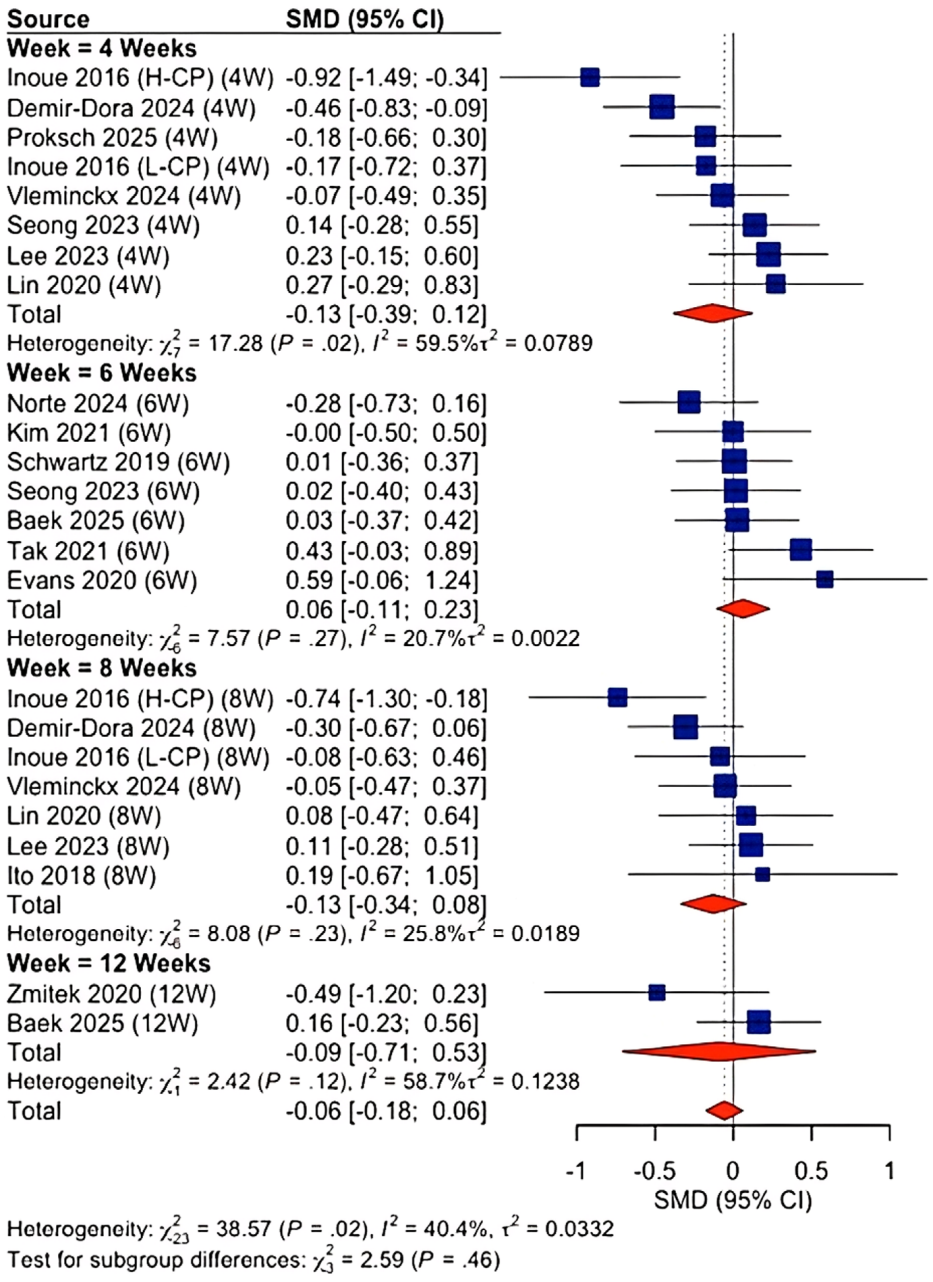
Forest plot showing standardized mean differences (SMD) with 95% confidence intervals (CIs) comparing the intervention group in different time stamps with controls for skin wrinkles.

In stark contrast to the findings for hydration and elasticity, the meta‐analysis of skin wrinkles demonstrated a complete lack of therapeutic efficacy across all time points. The initial assessment appeared to show a trend at week 8; however, this was entirely driven by a single extreme outlier, Proksch 2025, which reported a massive effect size (SMD = −3.70), contradicting the rest of the cohort (Figure S4A). Following the removal of this high‐leverage study, the corrected analysis confirmed that the intervention was statistically indistinguishable from placebo, with a negligible overall pooled effect size of −0.06 (95% CI: −0.18 to 0.06, *p* = 0.46; Figure 7). Regarding heterogeneity, the sensitivity analysis was validated by a dramatic reduction in statistical inconsistency: excluding the outlier dropped the Week 8 heterogeneity from a critical *I* ^2^ = 79.2%, *τ* = 0.4033 to a highly stable *I* ^2^ = 40.4%, *τ* = 0.0332, confirming that the initial variance was an artifact of data quality rather than true clinical diversity. Additionally, the majority of the studies included may have limited the scope of their analysis by focusing on 8–12 week protocols, which may have been too short to fully assess the potential for structural dermal remodeling, and the absence of any observed changes in wrinkles should be interpreted accordingly.

#### 3.2.5 Skin Roughness

**FIGURE 8:**
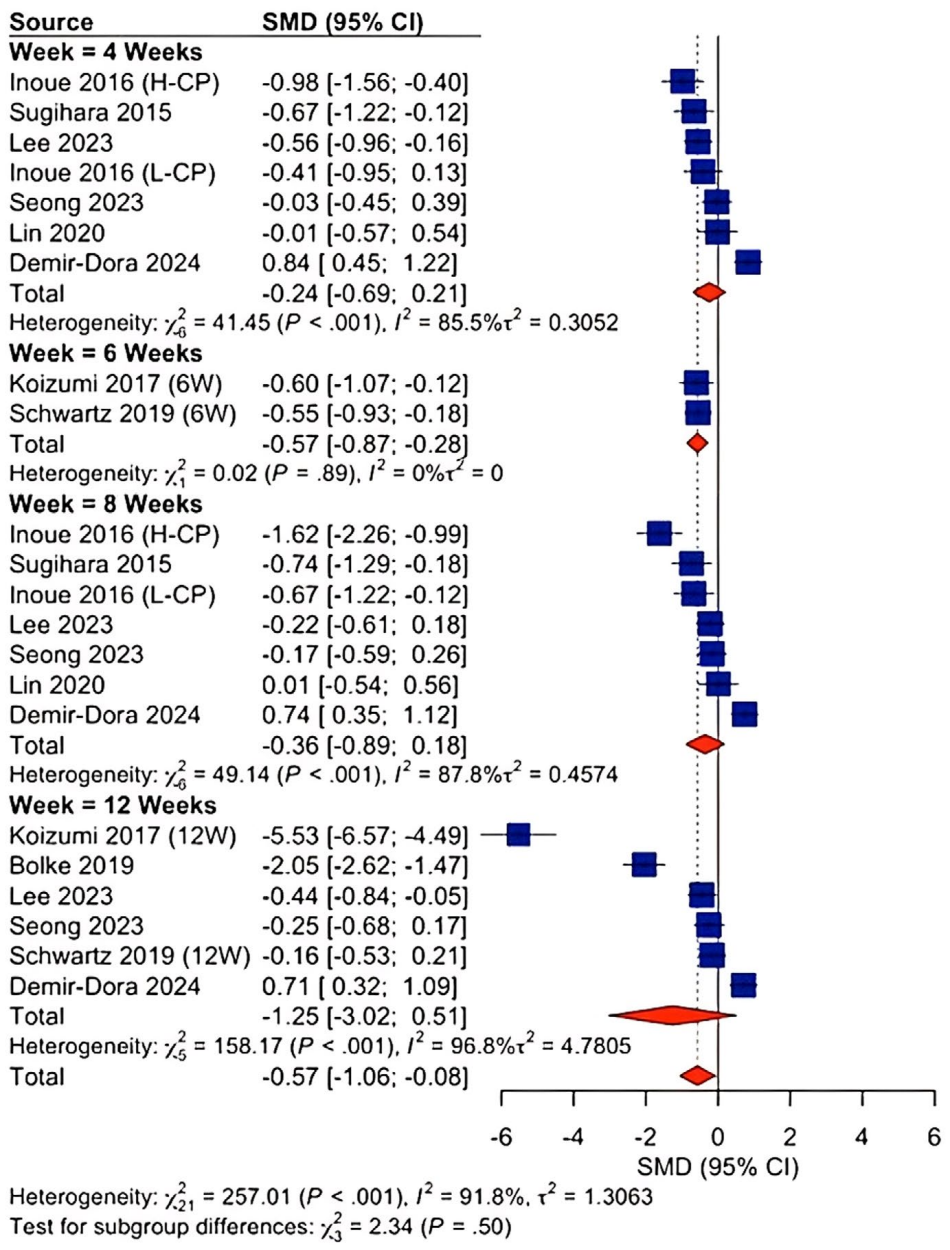
Forest plot showing standardized mean differences (SMD) with 95% confidence intervals (CIs) comparing the intervention group with controls in different time stamps for skin roughness.

The meta‐analysis of skin roughness demonstrated a statistically significant reduction in roughness scores (SMD = −0.57; 95% CI: −1.06 to −0.08; *p* = 0.02; Figure 8), although the estimate was complicated by extreme heterogeneity (*I* ^2^ = 91.8%). The subgroup analysis was significant at week 6 (SMD = −0.57, 95% CI: −0.87 to −0.28; Figure 8), whereas no significant improvements were observed at week 4 (SMD = −0.24, 95% CI: −0.69 to 0.21; Figure 8) or week 8 (SMD = −0.36, 95% CI: −0.89 to 0.18; Figure 8). Sensitivity analysis confirmed the statistical robustness of the findings; the results remained significant across all leave‐one‐out iterations (Figure S5B). Omitting Koizumi 2017 (12 weeks) nearly halved the effect size (SMD attenuated to −0.35), while excluding the contradictory positive data from Demir‐Dora 2024 strengthened the benefit (SMD increased to −0.64). Despite these exclusions, heterogeneity remained persistently high (*I* ^2^ > 87% in all scenarios), suggesting that the variability is systemic to the dataset and not driven by a single outlier.

#### 3.2.6 Skin Density

**FIGURE 9:**
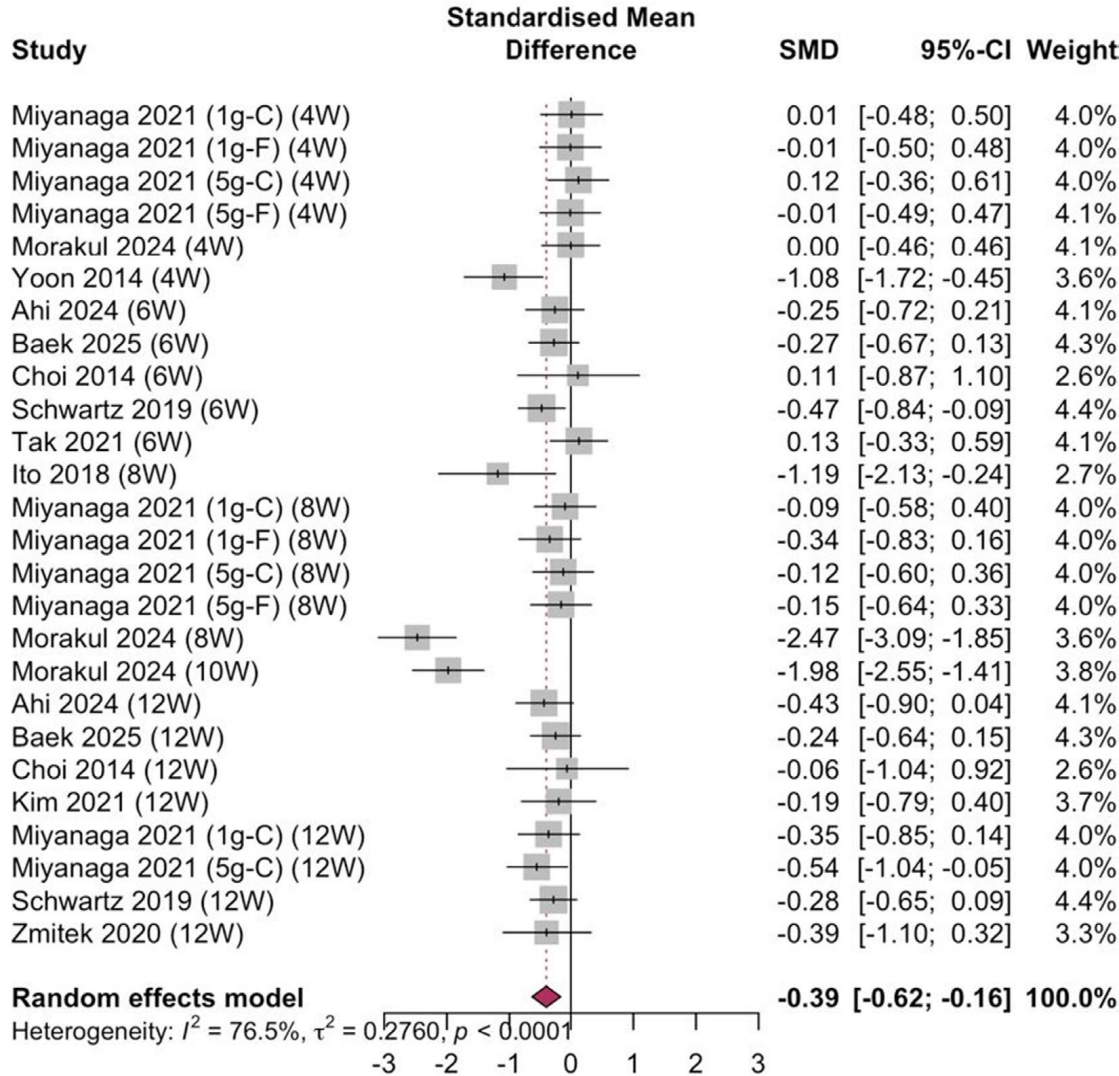
Forest plot showing standardized mean differences (SMD) with 95% confidence intervals (CIs) comparing the intervention group with controls in different time stamps for skin density.

The initial pooled analysis for skin density indicated a statistically significant reduction compared with the control group (SMD = −0.39; 95% CI: −0.62 to −0.16; *p* < 0.0001; Figure 9). However, this estimate was characterized by substantial statistical heterogeneity (*I* ^2^ = 76.5%, *τ* ^2^ = 0.2760; *p* < 0.0001). Visual inspection of the forest plot revealed wide variation in effect sizes, with several studies clustering around a null effect (e.g., [35]; Miyanaga 2021), while high‐leverage outliers, most notably Morakul et al. [14] and Ito et al. [50], contributed disproportionately to the negative effect size. Subsequent robustness checks, including a Multilevel Meta‐Analysis, shifted the estimate considerably (SMD = 0.64, 95% CI: −0.43 to 1.71), indicating that the primary negative significance was likely a statistical artifact driven by heterogeneity rather than a true clinical reduction. Consequently, the data do not support a significant therapeutic benefit or change in dermal density.

### 3.3 Sensitivity Analysis and Robustness Analysis

Strict robustness tests were necessary to differentiate actual clinical signals from statistical noise due to high‐leverage studies. The same finding was supported by a Multilevel Meta‐Analysis that indicated a stronger effect size (SMD = 0.81, CI = 0.19 to 1.42; Figure S2J) than the standard model on skin elasticity. Likewise, in the case of TEWL, the Multilevel model gave a similar estimate (SMD = −0.32, CI = −0.63 to −0.02; Figure S3E), which is practically the same as the primary analysis; this supports the fact that the benefit of Week 12 is not sensitive to model specification.

In the case of wrinkles, the leave‐one‐out analysis (Figure S4B) found that the only source of unsteadiness was Proksch 2025, and the multilevel model estimate (SMD = −0.02, 95% CI = −0.23 to 0.20; Figure S4N) clearly indicated that there was no hidden efficacy. To create the roughness, diagnostic plots showed Koizuni 2017 to be a disproportionate influencer; when they were dropped, the results became insignificant. Although the multilevel estimate (SMD = −1.09, 95% CI = −2.14 to −0.04; Figure S5K) retained a confidence interval that crossed zero.

The original positive result for skin density was disproved by robustness checks. Although the estimates in the leave‐one‐out sensitivity were similar (−0.34 to −0.15; Figure S6B), the Multilevel Meta‐Analysis indicated a considerable estimate (SMD 0.64, 95% CI −0.43 to 1.71; Figure S6V), which indicated that the primary significance was probably a Type I error. Multilevel meta‐analysis supported the strength of the effect of collagen in decreasing TEWL with a pooled SMD of −0.32 (95% CI = −0.63 to −0.02; Figure S3E), which is highly congruent with the conventional model and confirms a true Week 12 benefit. The leave‐one‐out analysis ensured that the estimates were stable (−1.06 to −0.08; Figure S3A), although the Morakul 2024 outlier had a downward effect (Table S1).

### 3.4 Subgroup Analysis and Meta‐Regression

The exploration of possible moderators, that is, dosage, duration, age, and baseline status, showed that length of stay is the key to efficacy and not demographic or dosing factors (Figures S1G, S1H, S1N, S2H, S2I, S3F, S3I, S3O, S3P, S3Q, S4H, S4L, S4M, S5F, S5H, S5L, S5M, S5O, S6G, S6H, S6K, S6P, and S6U). Time was the most predictive measure of success during the intake period. Hydration had a non‐linear curve that reached its peak at Week 4, whereas elasticity and TEWL (Figure 6) benefits were long‐term and only showed signs at Week 12. Duration did not have any linear relationship with roughness or density (Figures S5H and S6P). Meta‐regression showed no significant linear relationship between improvement and daily dosage (grams/day), with significance either way with hydration or roughness (*p* > 0.05) (Figures S1G and S5L). In the case of skin density (Figure S6G), elasticity, and TEWL (Figure S3O), dose–response spline regressions showed a non‐linear, chaotic interaction with a widening confidence interval at higher dosages, which did not show any support that higher dosage enhanced performance (Figures S1N, S3I, S4H, and S6H). Therefore, the only known predictor of success is the duration of intake. Baseline skin condition and mean age did not significantly predetermine the treatment outcomes in any case, which may indicate that the intervention is effective in either case of initial skin severity or participant age.

### 3.5 Publication Bias

Statistical tests showed a widespread publication bias throughout the dataset. In the case of Hydration & Elasticity, the Trim‐and‐Fill analysis revealed asymmetric funnel plots (Figures S1B, S3V, S4T, S5N, and S6D), which indicated that smaller negative studies might be omitted, implying that the effect sizes were slightly overstated. The Contour‐Enhanced Funnel Plots and Vevea and Woods results of Density and TEWL were very asymmetric and had a distinct lack of non‐significant studies, further showing that there can be an underreporting of the finding of no effect. Although asymmetry existed in Wrinkles & Roughness, when the missing studies were imputed, the adjusted effect sizes were still non‐significant, which proves that the failure of the intervention in the above outcomes is not a result of publication bias (Figures S1C, S3G, S4C, and S6C).

### 3.6 Clinical Impact Summary

Cumulative evidence favors a given therapeutic profile over time of intervention. The intervention had a large positive impact on physiological skin parameters, including hydration (SMD = 0.44; Figure 4), elasticity (SMD = 0.62; Figure 5), and barrier function/TEWL (SMD = −0.39; Figure 6). It did not offer structural advantages on wrinkles or skin density (Figures 7 and 9), where apparent initial effects were shown to be statistical artifacts facilitated by outliers and heterogeneity. For most outcomes, efficacy seemed to be more time than dose dependent, as effects on hydration were seen as early as week 4, while the elasticity and barrier reinforcement were more likely to require at least 12 weeks of supplementation. This pattern was more prominent than individual studies and outcome domain. Strength tests (multilevel models and sensitivity tests) validated that, whereas the physiological gains were real, the cosmetic gains (roughness, density, and wrinkles) were either null or not statistically significant, which means that the intervention is a functional moisturizer but not a structural anti‐aging agent.

## 4 Discussion

This expanded evidence synthesis indicates that oral collagen peptide supplementation results in statistically significant improvements across multiple cutaneous aging parameters. Looking across both the earlier literature and more recently conducted trials, the clearest and most reproducible benefits of oral collagen supplementation were observed for skin hydration, elasticity, and barrier function. The findings for wrinkle formation and dermal structural integrity, however, proved far less reliable—initial signals in these domains did not survive sensitivity testing, and upon deeper examination, appeared to reflect the disproportionate influence of statistical outliers and high inter‐study variability rather than any genuine therapeutic effect. These findings build upon earlier randomized investigations [40, 46, 49] while integrating newer studies characterized by enhanced methodological rigor and advanced imaging modalities.

Hydration and elasticity demonstrated the most stable effects, yielding relatively homogenous estimates across trials, and remained robust under diverse analytical conditions [26, 28, 30]. TEWL reductions, documented in controlled studies employing validated barrier function metrics [35], suggest that collagen peptides may influence epidermal physiology beyond aesthetic endpoints. While some individual primary studies have previously suggested potential improvements in dermal structural integrity [39, 43], our robust pooled analysis, which accounted for severe inter‐study heterogeneity and influential outliers, failed to demonstrate a statistically significant increase in overall dermal density. This suggests that the structural benefits of collagen peptides may be less pronounced than the physiological improvements in hydration and barrier function.

Mechanistic studies provide corroborative biological support. Studies on collagen‐related dipeptides have consistently shown stimulation of fibroblast activity, augmentation of type I collagen synthesis, and attenuation of matrix metalloproteinase activity [45, 50]. More recent molecular analyses have identified the concurrent regulation of proteoglycans and procollagen, as well as the suppression of degradative enzymatic pathways [44]. This mechanistic alignment strengthens the plausibility of the observed clinical effects and suggests a multifaceted impact on dermal remodeling.

The discrepancy between physiological improvements (hydration and elasticity) and the lack of structural changes (wrinkles) can be explained by the timeline of dermal tissue remodeling. While oral dipeptides can rapidly stimulate hyaluronic acid synthesis in the epidermis to acutely improve hydration and barrier function, the gross reduction of visible wrinkles requires extensive and long‐term deposition and reorganization of the dermal collagen matrix. It is highly probable that the follow‐up durations of the included trials (primarily 8 to 12 weeks) are sufficient to capture hydration shifts but insufficient to capture macroscopic reductions in wrinkle depth [8, 51].

Key differences between this analysis and the 2025 meta‐analysis [48] help to clarify why the conclusions diverge. First, our evidence base includes several randomized trials published after 2024 [26, 28, 29, 30, 31, 52], which were unavailable to the earlier authors and collectively exerted a significant influence on pooled estimates. Second, our analytic approach explicitly addressed formulation heterogeneity variations in molecular weight, amino acid sequence distribution, peptide origin, and dose, which may influence bioavailability and clinical outcomes. The prior review aggregated these heterogeneous products into a single exposure category, potentially attenuating formulation‐specific effects. Third, we applied a domain‐level RoB 2.0 assessment, which permitted a more discriminating evaluation of methodological limitations than the previously used dichotomous classification.

Concerns regarding the influence of industry funding have also been examined. Independently conducted trials [44, 48] reported effect sizes comparable to those observed in sponsored studies, and multiple approaches to detecting publication bias suggested that preferential reporting was unlikely to meaningfully alter the overall conclusions.

By synthesizing a broader range of randomized evidence and integrating mechanistic insights, this updated analysis provides a comprehensive and contemporary evaluation of collagen peptides as a dermatologic intervention. The remaining limitations of the current evidence base including considerable heterogeneity in collagen formulations, variability in outcome measurement methodologies, and the predominance of short‐term trial designs should temper the interpretation of findings, particularly for structural outcomes. That said, the consistency of improvements observed across hydration, elasticity, and barrier function domains, and their reproducibility across multiple analytical strategies does provide reasonable confidence in these specific physiological findings.

## 5 Conclusion

This meta‐analysis demonstrates that oral collagen peptide supplementation most reliably improves skin hydration and barrier function, with elasticity benefits emerging primarily at 12 weeks. Structural outcomes including wrinkle reduction and dermal densification did not demonstrate robust effects upon sensitivity analysis, with apparent benefits attributable to statistical artifacts rather than genuine treatment effects. These findings should be interpreted cautiously given the considerable heterogeneity in collagen formulations, outcome measures, and the predominantly short trial durations across included studies. Future research should prioritize standardized formulations, harmonized outcome measures, and longer follow‐up periods of at least 24 weeks to fully evaluate the structural anti‐aging potential of oral collagen supplementation.

## Author Contributions

Asia Batool generated the idea, Makhzan Ali Akbar and Asia Batool did screening, Muhammad Sharjeel Abbas did analysis, Ramzan Farooq, Ashfaq Ahmad and Asia Batool extracted data and made baselines table, Muhammad Junaid wrote results, Hasibullah Aminpoor and Muhammad Suhaib Hanif wrote introduction and methodology, Ashfaq Ahmad wrote discussion, Alisha Ahmed did ROB assessment, Warisha Kanwal wrote abstract, made graphical abstract, did referencing and compiled the manuscript. Evardo Barros de Deus Nunes Junior supervised the project.

## Funding

The authors have nothing to report.

## Ethics Statement

The authors have nothing to report.

## Consent

The authors have nothing to report.

## Conflicts of Interest

The authors declare no conflicts of interest.

## Supporting information


**Table S1:** Certainty assessment.
**Figure S1A:** Skin hydration meta analysis with outliers.
**Figure S1B:** Trim and fill analysis for skin hydration.
**Figure S1C:** Contour‐enhanced funnel plot for skin hydration.
**Figure S1D:** Influence plots for skin hydration.
**Figure S1E:** Baujat plot for skin hydration.
**Figure S1F:** Galbraith (radial) plot (skin hydration).
**Figure S1G:** Meta‐regression plots daily dose effect (skin hydration).
**Figure S1H:** Meta‐regression plots baseline hydration score (raw score).
**Figure S1I:** Forest plot with prediction interval (skin hydration).
**Figure S1J:** Gosh plot for skin hydration.png.
**Figure S1K:** Robust variance estimation (RVE). RVE comparison plot (skin hydration—red blue).
**Figure S1L:** Cumulative meta‐analysis (skin hydration).
**Figure S1M:** Comparative density plot (hydration vs. TEWL).
**Figure S1N:** Dose–response spline regression (skin hydration).
**Figure S1O:** Dumbbell plot (skin hydration).
**Figure S1P:** L'Abb plot (skin hydration).
**Figure S1Q:** Raincloud plot (skin hydration).
**Figure S1R:** Skin hydration/impact plot *p*‐value function.
**Figure S1S:** Spaghetti plot (skin hydration).
**Figure S1T:** Vevea & Woods custom plot for skin hydration.
**Figure S2A:** Main meta‐analysis skin elasticity with outliers.
**Figure S2B:** Egger's test & funnel plot (skin elasticity).
**Figure S2C:** Influence plots (skin elasticity).
**Figure S2D:** Forest plot with prediction interval (skin elasticity).
**Figure S2E:** Cumulative meta‐analysis (skin elasticity).
**Figure S2F:** Galbraith (radial) plot (skin elasticity).
**Figure S2G:** Gosh analysis for skin elasticity.
**Figure S2H:** Meta regression baseline elasticity score.
**Figure S2I:** Meta regression mean age for skin elasticity.
**Figure S2J:** Multilevel forest plot (skin elasticity).
**Figure S3A:** TEWL influence plots leave one out.
**Figure S3B:** RVE comparison plot (TEWL).
**Figure S3C:** Cumulative meta‐analysis (TEWL).
**Figure S3D:** RVE comparison plot (TEWL).
**Figure S3E:** Multilevel meta‐analysis for TEWL.
**Figure S3F:** Meta reg baseline TEWL score (~ Baseline_Mean).
**Figure S3G:** Contour‐enhanced funnel plot (TEWL).
**Figure S3H:** Comparative density plot (hydration vs. TEWL).
**Figure S3I:** Dose–response spline regression (TEWL).
**Figure S3J:** Dumbbell plot (TEWL).
**Figure S3K:** Egger's test & funnel plot (TEW).
**Figure S3L:** Gosh analysis for TEWl.
**Figure S3M:** Impact plot (TEWL).
**Figure S3N:** L'Abb plot (TEWL).
**Figure S3O:** Meta reg for (TEWL) daily dosage (~ Dosage).
**Figure S3P:** Meta reg for (TEWL) mean age (~ Mean_Age).
**Figure S3Q:** Meta reg for (TEWL) duration of supplementation (~ Duration).
**Figure S3R:** Prediction interval forest plot (TEWL).
**Figure S3S:** Raincloud plot (TEWL).
**Figure S3T:** Spaghetti plot (TEWL).
**Figure S3U:** TEWL Galbraith plots.
**Figure S3V:** Trim and fill analysis (TEWL—cleaned data).
**Figure S3W:** Vevea & Woods custom plot (TEWL).
**Figure S4A:** Main meta‐analysis for skin wrinkles with outliers.
**Figure S4B:** Influence plot (skin wrinkles). Leave one out.
**Figure S4C:** Contour‐enhanced funnel plot (skin wrinkles).
**Figure S4D:** L'Abb plot (skin wrinkles).
**Figure S4E:** Cumulative meta‐analysis (skin wrinkles).
**Figure S4F:** Baujat (skin wrinkles).
**Figure S4G:** Comparative density plot (wrinkles vs. density).
**Figure S4H:** Dose–response spline regression (skin wrinkles).
**Figure S4I:** Egger's test & funnel plot (skin wrinkles).
**Figure S4J:** Galbraith plots (skin wrinkles).
**Figure S4K:** Impact plot (skin wrinkles).
**Figure S4L:** Mean age of participants (wrinkles) meta regression.
**Figure S4M:** Meta regression baseline wrinkle score.
**Figure S4N:** Multilevel forest plot (wrinkles).
**Figure S4O:** Prediction interval (skin wrinkles).
**Figure S4P:** Raincloud plot (skin wrinkles).
**Figure S4Q:** RVE comparison plot (skin wrinkles).
**Figure S4R:** Skin wrinkles Dumbbell plot.
**Figure S4S:** Spaghetti plot (skin wrinkles).
**Figure S4T:** Trim and fill analysis (skin wrinkles).
**Figure S4U:** Vevea & Woods custom plot (skin wrinkles).
**Figure S5A:** Egger's test & funnel plot (skin roughness).
**Figure S5B:** Influence plots (skin roughness).
**Figure S5C:** Robust variance estimation (RVE). RVE comparison plot (skin hydration—red blue).
**Figure S5D:** Prediction interval (skin roughness) forest plot.
**Figure S5E:** Cumulative meta‐analysis (skin roughness).
**Figure S5F:** Baseline roughness score (floor effect test).
**Figure S5G:** Baujat plots (skin roughness).
**Figure S5H:** Duration of supplementation (skin turnover cycle) roughness meta‐regression plots.
**Figure S5I:** Galbraith plots (skin roughness).
**Figure S5J:** Gosh analysis for skin roughness.
**Figure S5K:** Multilevel forest plot (roughness).
**Figure S5L:** Roughness meta‐regression plots daily dosage (grams day).
**Figure S5M:** Roughness meta‐regression plots mean age of participants.
**Figure S5N:** Trim and fill analysis (skin roughness).
**Figure S5O:** Baseline roughness score (floor effect test).
**Figure S6A:** Egger's test & funnel plot (skin density).
**Figure S6B:** Influence plots (skin density—leave one out meta analysis).
**Figure S6C:** Contour‐enhanced funnel plot (skin density).
**Figure S6D:** Trim and fill analysis (skin density).
**Figure S6E:** Vevea & Woods custom plot (skin density).
**Figure S6F:** Egger's test & funnel plot (skin density).
**Figure S6G:** Meta Reg for skin density daily dosage (~ Dosage).
**Figure S6H:** Dose–response spline regression for the skin density.
**Figure S6I:** Cumulative meta‐analysis (skin density—blue).
**Figure S6J:** Prediction interval forest plot (skin density).
**Figure S6K:** Baseline density score (~ Baseline_Mean).
**Figure S6L:** Baujat plots (skin density).
**Figure S6M:** Comparative density plot (hydration vs. TEWL).
**Figure S6N:** Comparative density plot (wrinkles vs. density).
**Figure S6O:** Dumbbell plot (skin density).
**Figure S6P:** Duration of supplementation (~ Duration) meta regression for skin density.
**Figure S6Q:** Galbraith plots (skin density).
**Figure S6R:** Gosh analysis for skin density.
**Figure S6S:** Impact plot (skin density).
**Figure S6T:** L'Abbé plot (skin density).
**Figure S6U:** Meta regression for skin density mean age (~ Mean_Age).
**Figure S6V:** Multilevel meta‐analysis with plot (skin density—blue).
**Figure S6W:** Raincloud plot (skin density).
**Figure S6X:** Spaghetti plot (skin density).
**Figure S6Y:** Denser multi‐track circos plot (source, region, efficacy).

## Data Availability

The data that support the findings of this study are available from the corresponding author upon reasonable request.
