# Supplementary material for: Oral Collagen Peptides and Skin Rejuvenation: A Systematic Review and an Updated Meta‐Analysis of Randomized Controlled Trials
Source: J Cosmet Dermatol. 2026 Jul 15;25(7):e71041. doi: 10.1111/jocd.71041 (PMC13370842; doi:10.1111/jocd.71041)
Supplement: Supplementary file 1 — Table S1: Certainty assessment. Figure S1A: Skin hydration meta analysis with outliers. Figure S1B: Trim and fill analysis for skin hydration. Figure S1C: Contour‐enhanced funnel plot for skin hydration. Figure S1D: Influence plots for skin hydration. Figure S1E: Baujat plot for skin hydration. Figure S1F: Galbraith (radial) plot (skin hydration). Figure S1G: Meta‐regression plots daily dose effect (skin hydration). Figure S1H: Meta‐regression plots baseline hydration score (raw score). Figure S1I: Forest plot with prediction interval (skin hydration). Figure S1J: Gosh plot for skin hydration.png. Figure S1K: Robust variance estimation (RVE). RVE comparison plot (skin hydration—red blue). Figure S1L: Cumulative meta‐analysis (skin hydration). Figure S1M: Comparative density plot (hydration vs. TEWL). Figure S1N: Dose–response spline regression (skin hydration). Figure S1O: Dumbbell plot (skin hydration). Figure S1P: L'Abb plot (skin hydration). Figure S1Q: Raincloud plot (skin hydration). Figure S1R: Skin hydration/impact plot p‐value function. Figure S1S: Spaghetti plot (skin hydration). Figure S1T: Vevea & Woods custom plot for skin hydration. Figure S2A: Main meta‐analysis skin elasticity with outliers. Figure S2B: Egger's test & funnel plot (skin elasticity). Figure S2C: Influence plots (skin elasticity). Figure S2D: Forest plot with prediction interval (skin elasticity). Figure S2E: Cumulative meta‐analysis (skin elasticity). Figure S2F: Galbraith (radial) plot (skin elasticity). Figure S2G: Gosh analysis for skin elasticity. Figure S2H: Meta regression baseline elasticity score. Figure S2I: Meta regression mean age for skin elasticity. Figure S2J: Multilevel forest plot (skin elasticity). Figure S3A: TEWL influence plots leave one out. Figure S3B: RVE comparison plot (TEWL). Figure S3C: Cumulative meta‐analysis (TEWL). Figure S3D: RVE comparison plot (TEWL). Figure S3E: Multilevel meta‐analysis for TEWL. Figure S3F: Meta reg baseline TEWL score (~ Baseline_Mea [file JOCD-25-e71041-s001.docx]

**SUPPLEMENTAL APPENDIX**

**For**

**“Oral Collagen Peptides and Skin Rejuvenation: A Systematic Review and An Updated Meta-Analysis of Randomized Controlled Trials”**

**PRISMA Checklist**

| **Section and Topic** | **Item #** | **Checklist item** | **Location where item is reported** |
| --- | --- | --- | --- |
| **TITLE** | | |  |
| Title | 1 | Identify the report as a systematic review. | Page 1 |
| **ABSTRACT** | | |  |
| Abstract | 2 | See the PRISMA 2020 for Abstracts checklist. | Page 3 |
| **INTRODUCTION** | | |  |
| Rationale | 3 | Describe the rationale for the review in the context of existing knowledge. | Page 4-6 |
| Objectives | 4 | Provide an explicit statement of the objective(s) or question(s) the review addresses. | Page 6 |
| **METHODS** | | |  |
| Eligibility criteria | 5 | Specify the inclusion and exclusion criteria for the review and how studies were grouped for the syntheses. | Page 7-8 |
| Information sources | 6 | Specify all databases, registers, websites, organisations, reference lists and other sources searched or consulted to identify studies. Specify the date when each source was last searched or consulted. | Page 6 |
| Search strategy | 7 | Present the full search strategies for all databases, registers and websites, including any filters and limits used. | Page 6-7 |
| Selection process | 8 | Specify the methods used to decide whether a study met the inclusion criteria of the review, including how many reviewers screened each record and each report retrieved, whether they worked independently, and if applicable, details of automation tools used in the process. | Page 7 |
| Data collection process | 9 | Specify the methods used to collect data from reports, including how many reviewers collected data from each report, whether they worked independently, any processes for obtaining or confirming data from study investigators, and if applicable, details of automation tools used in the process. | Page 8 |
| Data items | 10a | List and define all outcomes for which data were sought. Specify whether all results that were compatible with each outcome domain in each study were sought (e.g. for all measures, time points, analyses), and if not, the methods used to decide which results to collect. | Page 7 |
|  | 10b | List and define all other variables for which data were sought (e.g. participant and intervention characteristics, funding sources). Describe any assumptions made about any missing or unclear information. | Page 7, Table 1 |
| Study risk of bias assessment | 11 | Specify the methods used to assess risk of bias in the included studies, including details of the tool(s) used, how many reviewers assessed each study and whether they worked independently, and if applicable, details of automation tools used in the process. | Page 8 |
| Effect measures | 12 | Specify for each outcome the effect measure(s) (e.g. risk ratio, mean difference) used in the synthesis or presentation of results. | Page 8 |
| Synthesis methods | 13a | Describe the processes used to decide which studies were eligible for each synthesis (e.g. tabulating the study intervention characteristics and comparing against the planned groups for each synthesis (item #5)). | Page 9 |
|  | 13b | Describe any methods required to prepare the data for presentation or synthesis, such as handling of missing summary statistics, or data conversions. | Page 9 |
|  | 13c | Describe any methods used to tabulate or visually display results of individual studies and syntheses. | Page 9 |
|  | 13d | Describe any methods used to synthesize results and provide a rationale for the choice(s). If meta-analysis was performed, describe the model(s), method(s) to identify the presence and extent of statistical heterogeneity, and software package(s) used. | Page 9 |
|  | 13e | Describe any methods used to explore possible causes of heterogeneity among study results (e.g. subgroup analysis, meta-regression). | Page 9 |
|  | 13f | Describe any sensitivity analyses conducted to assess robustness of the synthesized results. | Page 9 |
| Reporting bias assessment | 14 | Describe any methods used to assess risk of bias due to missing results in a synthesis (arising from reporting biases). | Page 9 |
| Certainty assessment | 15 | Describe any methods used to assess certainty (or confidence) in the body of evidence for an outcome. | Page 9 |
| **RESULTS** | | |  |
| Study selection | 16a | Describe the results of the search and selection process, from the number of records identified in the search to the number of studies included in the review, ideally using a flow diagram. | Page 9-10, Figure 1 |
|  | 16b | Cite studies that might appear to meet the inclusion criteria, but which were excluded, and explain why they were excluded. | Table 1 |
| Study characteristics | 17 | Cite each included study and present its characteristics. | Page 10  Table 1 |
| Risk of bias in studies | 18 | Present assessments of risk of bias for each included study. | Page 15 |
| Results of individual studies | 19 | For all outcomes, present, for each study: (a) summary statistics for each group (where appropriate) and (b) an effect estimate and its precision (e.g. confidence/credible interval), ideally using structured tables or plots. | Page 10-15 |
| Results of syntheses | 20a | For each synthesis, briefly summarise the characteristics and risk of bias among contributing studies. | Page 10-15 |
|  | 20b | Present results of all statistical syntheses conducted. If meta-analysis was done, present for each the summary estimate and its precision (e.g. confidence/credible interval) and measures of statistical heterogeneity. If comparing groups, describe the direction of the effect. | Page 10-15 |
|  | 20c | Present results of all investigations of possible causes of heterogeneity among study results. | Page 10-15 |
|  | 20d | Present results of all sensitivity analyses conducted to assess the robustness of the synthesized results. | Page 10-15 |
| Reporting biases | 21 | Present assessments of risk of bias due to missing results (arising from reporting biases) for each synthesis assessed. | Page 15 |
| Certainty of evidence | 22 | Present assessments of certainty (or confidence) in the body of evidence for each outcome assessed. | Page 15 |
| **DISCUSSION** | | |  |
| Discussion | 23a | Provide a general interpretation of the results in the context of other evidence. | Page 16-18 |
|  | 23b | Discuss any limitations of the evidence included in the review. | Page 16-18 |
|  | 23c | Discuss any limitations of the review processes used. | Page 16-18 |
|  | 23d | Discuss implications of the results for practice, policy, and future research. | Page 16-18 |
| **OTHER INFORMATION** | | |  |
| Registration and protocol | 24a | Provide registration information for the review, including register name and registration number, or state that the review was not registered. | Page 19 |
|  | 24b | Indicate where the review protocol can be accessed, or state that a protocol was not prepared. | Page 19 |
|  | 24c | Describe and explain any amendments to information provided at registration or in the protocol. | Page 6 |
| Support | 25 | Describe sources of financial or non-financial support for the review, and the role of the funders or sponsors in the review. | Page 18 |
| Competing interests | 26 | Declare any competing interests of review authors. | Page 18 |
| Availability of data, code and other materials | 27 | Report which of the following are publicly available and where they can be found: template data collection forms; data extracted from included studies; data used for all analyses; analytic code; any other materials used in the review. | Page 19 |

**SEARCH STRING:**

Healthy[All Fields] AND ("skin"[MeSH Terms] OR skin[Text Word]) AND Collagen OR Collagen supplements AND "skin aging"[MeSH Terms] AND "elasticity"[MeSH Terms] OR Elasticity [Text Word] AND Randomised controlled trial

Pubmed: 1219

Search Name:

Date Run: 04/11/2025 03:27:42

ID Search Hits

#1 (Skin):ab (Word variations have been searched) 66089

#2 MeSH descriptor: [Collagen] explode all trees 3273

#3 MeSH descriptor: [Aged] explode all trees 286474

#4 MeSH descriptor: [Skin Aging] explode all trees 1318

#5 #1 AND #2 AND #3 AND #4 33

Cochrane:33

Healthy[All Fields] AND ("skin"[MeSH Terms] OR skin[Text Word]) AND Collagen OR Collagen supplements AND "skin aging"[MeSH Terms] AND "elasticity"[MeSH Terms] OR Elasticity[Text Word] AND Randomised controlled trial

Google scholar : 46

Duplicates:54

Primary screening:1244

Secondary screening:41

Included:35

Supplementary Table S1. Certainty assessment

| **Certainty assessment** | | | | | | | **№ of patients** | | **Effect** | | **Certainty** | **Importance** |
| --- | --- | --- | --- | --- | --- | --- | --- | --- | --- | --- | --- | --- |
| **№ of studies** | **Study design** | **Risk of bias** | **Inconsistency** | **Indirectness** | **Imprecision** | **Other considerations** | **Oral collagen peptides** | **Placebo** | **Relative (95% CI)** | **Absolute (95% CI)** |  |  |
| **Skin Hydration** | | | | | | | | | | | | |
| 35 | randomised trials | not serious | serious | not serious | not serious | none | 1312 | 1288 | - | SMD **0.78 SD higher** (0.23 higher to 1.33 higher) | ⨁⨁⨁◯ Moderate |  |
| **Skin Elasticity** | | | | | | | | | | | | |
| 35 | randomised trials | not serious | serious | not serious | not serious | none | 1312 | 1288 | - | SMD **0.62 SD higher** (0.15 higher to 1.1 higher) | ⨁⨁⨁◯ Moderate |  |
| **Skin Wrinkles** | | | | | | | | | | | | |
| 35 | randomised trials | not serious | not serious | not serious | serious | none | 1312 | 1288 | - | SMD **0.06 SD lower** (0.18 lower to 0.06 higher) | ⨁⨁⨁◯ Moderate |  |
| **Skin Roughness** | | | | | | | | | | | | |
| 35 | randomised trials | not serious | serious | not serious | not serious | none | 1312 | 1288 | - | SMD **0.57 SD lower** (1.06 lower to 0.08 lower) | ⨁⨁⨁◯ Moderate |  |
| **Trans epidermal water loss (TEWL)** | | | | | | | | | | | | |
| 35 | randomised trials | not serious | serious | not serious | not serious | none | 1312 | 1288 | - | SMD **0.39 SD lower** (0.62 lower to 0.16 lower) | ⨁⨁⨁◯ Moderate |  |
| **Skin density** | | | | | | | | | | | | |
| 35 | randomised trials | not serious | not serious | not serious | not serious | none | 1312 | 1288 | - | SMD **0.39 SD lower** (0.62 lower to 0.16 lower) | ⨁⨁⨁⨁ High |  |

**CI:** confidence interval; **SMD:** standardised mean difference

**Supplementary Figure S1A.** Skin Hydration Meta analysis with outliers

**Supplementary Figure S1B.** Trim and Fill Analysis for Skin Hydration

**Supplementary Figure S1C.** Contour-Enhanced Funnel Plot for Skin Hydration

**Supplementary Figure S1D.** Influence Plots for Skin Hydration

**Supplementary Figure S1E.** Baujat Plot for Skin Hydration

**Supplementary Figure S1F.** Galbraith (Radial) Plot (Skin Hydration)

**Supplementary Figure S1G.** Meta-Regression Plots daily dose effect (Skin Hydration)

**Supplementary Figure S1H.** Meta-Regression Plots Baseline Hydration Score (Raw Score)

**Supplementary Figure S1I.** Forest Plot with Prediction Interval (Skin Hydration)

**Supplementary Figure S1J.** Gosh plot for Skin Hydration.png

**Supplementary Figure S1K.** Robust Variance Estimation (RVE). RVE Comparison Plot (Skin Hydration - Red Blue)

**Supplementary Figure S1L.** Cumulative Meta-Analysis (Skin Hydration)

**Supplementary Figure S1M.** Comparative Density Plot (Hydration vs. TEWL)

**Supplementary Figure S1N.** Dose-Response Spline Regression (Skin Hydration )

**Supplementary Figure S1O.** Dumbbell Plot (Skin Hydration)

**Supplementary Figure S1P.** L'Abb Plot (Skin Hydration)

**Supplementary Figure S1Q.** Raincloud Plot (Skin Hydration)

**Supplementary Figure S1R.** Skin Hydration/ Impact Plot P-value Function

**Supplementary Figure S1S.**Spaghetti Plot (Skin Hydration)

**Supplementary Figure S1T.** Vevea & Woods Custom Plot for skin hydration.

**Supplementary Figure S2A.** Main Meta-Analysis Skin Elasticity with outliers

**Supplementary Figure S2B.** Egger's Test & Funnel Plot (Skin Elasticity)

**Supplementary Figure S2C.** Influence Plots (Skin Elasticity)

**Supplementary Figure S2D.** Forest Plot with Prediction Interval (Skin Elasticity)

**Supplementary Figure S2E.**Cumulative Meta-Analysis (Skin Elasticity)

**Supplementary Figure S2F.** Galbraith (Radial) Plot (Skin Elasticity)

**Supplementary Figure S2G.** Gosh Analysis for Skin Elasticity

**Supplementary Figure S2H.** Meta Regression Baseline Elasticity Score

**Supplementary Figure S2I.** Meta Regression Mean age for Skin Elasticity

**Supplementary Figure S2J.** Multilevel Forest Plot (Skin Elasticity)

**Supplementary Figure S3A.** TEWL Influence Plots Leave One Out

**Supplementary Figure S3B.** RVE Comparison Plot (TEWL).

**Supplementary Figure S3C.** Cumulative Meta-Analysis (TEWL

**Supplementary Figure S3D.** RVE Comparison Plot (TEWL).

**Supplementary Figure S3E.** Multilevel Meta-Analysis for TEWL

**Supplementary Figure S3F.** Meta Reg Baseline TEWL Score (~ Baseline_Mean)

**Supplementary Figure S3G.** Contour-Enhanced Funnel Plot (TEWL)

**Supplementary Figure S3H.** Comparative Density Plot (Hydration vs. TEWL)

**Supplementary Figure S3I.** Dose-Response Spline Regression (TEWL )

**Supplementary Figure S3J.** Dumbbell Plot (TEWL)

**Supplementary Figure S3K.** Egger's Test & Funnel Plot (TEW

**Supplementary Figure S3L.** Gosh Analysis for TEWl

**Supplementary Figure S3M.** Impact Plot (TEWL)

**Supplementary Figure S3N.** L'Abb Plot (TEWL )

**Supplementary Figure S3O.** Meta Reg for (TEWL) Daily Dosage (~ Dosage)

**Supplementary Figure S3P.** Meta Reg for (TEWL) Mean Age (~ Mean_Age)

**Supplementary Figure S3Q.** Meta Reg. for (TEWL) Duration of Supplementation (~ Duration)

**Supplementary Figure S3R.** Prediction Interval Forest Plot (TEWL)

**Supplementary Figure S3S.** Raincloud Plot (TEWL)

**Supplementary Figure S3T.** Spaghetti Plot (TEWL)

**Supplementary Figure S3U.** TEWL Galbraith Plots

**Supplementary Figure S3V.** Trim and Fill Analysis (TEWL - Cleaned Data)

**Supplementary Figure S3W.** Vevea & Woods Custom Plot (TEWL)

**Supplementary Figure S4A.** Main Meta-Analysis for Skin Wrinkles with outliers

**Supplementary Figure S4B.** Influence Plot (Skin Wrinkles). Leave one out

**Supplementary Figure S4C.** Contour-Enhanced Funnel Plot (Skin Wrinkles)

**Supplementary Figure S4D.** L'Abb Plot (Skin Wrinkles )

**Supplementary Figure S4E.** Cumulative Meta-Analysis (Skin Wrinkles)

**Supplementary Figure S4F.** Baujat (Skin Wrinkles)

**Supplementary Figure S4G.** Comparative Density Plot (Wrinkles vs. Density)

**Supplementary Figure S4H.** Dose-Response Spline Regression (Skin Wrinkles )

**Supplementary Figure S4I.** Egger's Test & Funnel Plot (Skin Wrinkles)

**Supplementary Figure S4J.** Galbraith Plots (Skin Wrinkles)

**Supplementary Figure S4K.** Impact Plot (Skin Wrinkles)

**Supplementary Figure S4L.** Mean Age of Participants (Wrinkles) Meta Regression

**Supplementary Figure S4M.** Meta Regression Baseline Wrinkle Score

**Supplementary Figure S4N.** Multilevel Forest Plot (Wrinkles)

**Supplementary Figure S4O.** Prediction Interval (Skin Wrinkles)

**Supplementary Figure S4P.** Raincloud Plot (Skin Wrinkles)

**Supplementary Figure S4Q.** RVE Comparison Plot (Skin Wrinkles)

**Supplementary Figure S4R.** Skin Wrinkles Dumbbell Plot

**Supplementary Figure S4S.** Spaghetti Plot (Skin Wrinkles)

**Supplementary Figure S4T.** Trim and Fill Analysis (Skin Wrinkles)

**Supplementary Figure S4U.** Vevea & Woods Custom Plot (Skin Wrinkles)

**Supplementary Figure S5A.** Egger's Test & Funnel Plot (Skin Roughness)

**Supplementary Figure S5B.** Influence Plots (Skin Roughness)

**Supplementary Figure S5C.** Robust Variance Estimation (RVE). RVE Comparison Plot (Skin Hydration - Red Blue)

**Supplementary Figure S5D.** Prediction Interval (Skin Roughness) Forest plot

**Supplementary Figure S5E.** Cumulative Meta-Analysis (Skin Roughness)

**Supplementary Figure S5F.** Baseline Roughness Score (Floor Effect Test)

**Supplementary Figure S5G.** Baujat Plots (Skin Roughness)

**Supplementary Figure S5H.** Duration of Supplementation (Skin Turnover Cycle) Roughness Meta-Regression Plots

**Supplementary Figure S5I.** Galbraith Plots (Skin Roughness )

**Supplementary Figure S5J.** Gosh Analysis For Skin roughness

**Supplementary Figure S5K.** Multilevel Forest Plot (Roughness)

**Supplementary Figure S5L.** Roughness Meta-Regression Plots Daily Dosage (Grams Day)

**Supplementary Figure S5M.** Roughness Meta-Regression Plots Mean Age of Participants

**Supplementary Figure S5N.** Trim and Fill Analysis (Skin Roughness)

**Supplementary Figure S5O.** Baseline Roughness Score (Floor Effect Test)

**Supplementary Figure S6A.** Egger's Test & Funnel Plot (Skin Density)

**Supplementary Figure S6B.** Influence Plots (Skin Density - Leave One Out Meta analysis )

**Supplementary Figure S6C.** Contour-Enhanced Funnel Plot (Skin Density)

**Supplementary Figure S6D.** Trim and Fill Analysis (Skin Density )

**Supplementary Figure S6E.** Vevea & Woods Custom Plot (Skin Density)

**Supplementary Figure S6F.** Egger's Test & Funnel Plot (Skin Density)

**Supplementary Figure S6G.** Meta Reg for Skin density Daily Dosage (~ Dosage)

**Supplementary Figure S6H.** Dose-Response Spline Regression for the Skin Density

**Supplementary Figure S6I.** Cumulative Meta-Analysis (Skin Density - Blue)

**Supplementary Figure S6J.** Prediction Interval Forest Plot (Skin Density )

**Supplementary Figure S6K.** Baseline Density Score (~ Baseline_Mean)

**Supplementary Figure S6L.** Baujat Plots (Skin Density )

**Supplementary Figure S6M.** Comparative Density Plot (Hydration vs. TEWL)

**Supplementary Figure S6N.** Comparative Density Plot (Wrinkles vs. Density)

**Supplementary Figure S6O.** Dumbbell Plot (Skin Density)

**Supplementary Figure S6P.** Duration of Supplementation (~ Duration) Meta regression for skin density

**Supplementary Figure S6Q.** Galbraith Plots (Skin Density )

**Supplementary Figure S6R.** Gosh analysis for skin density

**Supplementary Figure S6S.** Impact Plot (Skin Density)

**Supplementary Figure S6T.** L'Abbé Plot (Skin Density )

**Supplementary Figure S6U.** Meta Regression for Skin density Mean Age (~ Mean_Age)

**Supplementary Figure S6V.** Multilevel Meta-Analysis with Plot (Skin Density - Blue)

**Supplementary Figure S6W.** Raincloud Plot (Skin Density)

**Supplementary Figure S6X.** Spaghetti Plot (Skin Density)

**Supplementary Figure S6Y.** Denser Multi-Track Circos Plot (Source, Region, Efficacy)
